# The impact of psoriatic arthritis on quality of life: a systematic review

**DOI:** 10.1177/1759720X241295920

**Published:** 2024-12-22

**Authors:** Lija James, Louise H. Hailey, Rhea Suribhatla, Dylan McGagh, Raj Amarnani, Christine E. Bundy, Shona Kirtley, Denis O’Sullivan, Ingrid Steinkoenig, Jonathan P. E. White, Arani Vivekanantham, Laura C. Coates

**Affiliations:** Nuffield Department of Orthopaedics Rheumatology and Musculoskeletal Sciences, University of Oxford, Oxford, UK; Nuffield Department of Orthopaedics Rheumatology and Musculoskeletal Sciences, University of Oxford, Oxford, UK; Oriel College, University of Oxford, Oxford, UK; Nuffield Department of Orthopaedics Rheumatology and Musculoskeletal Sciences, University of Oxford, Oxford, UK; Barts Health NHS Trust, London, UK; Behavioural Medicine/Health Psychology School of Healthcare Sciences, Cardiff University, Cardiff, UK; Centre for Statistics in Medicine, University of Oxford, Oxford, UK; Group for Research and Assessment of Psoriasis and Psoriatic Arthritis, Patient Research Partner, Kildare, Ireland; Group for Research and Assessment of Psoriasis and Psoriatic Arthritis, Patient Research Partner, Cleveland, OH, USA; Department of Dermatology, University Hospitals Sussex NHS Foundation Trust, Worthing, UK; Nuffield Department of Orthopaedics Rheumatology and Musculoskeletal Sciences, University of Oxford, Oxford, UK; Nuffield Department of Orthopaedics, Rheumatology and Musculoskeletal Sciences, Botnar Research Centre, Windmill Road, Oxford OX3 7HE, UK

**Keywords:** health-related quality of life, patient-reported outcomes, psoriatic arthritis

## Abstract

**Background:**

Psoriatic arthritis (PsA) is a chronic inflammatory condition that can affect individuals of all ages. Patients may experience a range of physical and psychological issues.

**Objective:**

To examine the impact of PsA on an individual’s quality of life (QoL) and physical function.

**Design:**

A systematic review of the literature.

**Data sources and methods:**

A comprehensive search was conducted across seven electronic databases (Cochrane Central Register of Controlled Trials (CENTRAL), CINAHL, AMED, EMBASE, Global Health, MEDLINE and PsycINFO) to retrieve articles related to QoL and lifestyle in adults with PsA. The inclusion criteria were studies published between 2010 and 2021 that used outcomes validated in patients with PsA. The methodological quality was assessed using Joanna Briggs Institute Critical Appraisal Tools. Our primary outcomes were patient-reported outcomes (PROs) measuring QoL and the impact of disease on physical function. The secondary outcomes were assessments of fatigue, anxiety, depression, sleep, work productivity and employment.

**Results:**

The study included 37 comprehensive studies that examined the impact of PsA on QoL and physical function. The findings revealed that the impact of PsA extends to various aspects of life, including activities of daily living, physical, and emotional aspects, such as fatigue, sleep disturbance, anxiety and depression. Notably, people with PsA experience reduced health-related quality of life (HRQoL), particularly in emotional, social and mental health aspects. The severity of pain and/or fatigue is directly linked to decreased HRQoL. Importantly, those who fail to achieve minimal disease activity face challenges in work productivity and employment status.

**Conclusion:**

To conclude, our review underscores the significant impact of PsA on patients’ HRQoL beyond joint disease. The emotional, social, and mental aspects of PsA require compassionate and holistic management.

**Trial registration:**

The PROSPERO international prospective register of systematic reviews – CRD42021257395.

## Plain language summary

The impact of psoriatic arthritis on quality of life

Psoriatic arthritis (PsA) is a type of joint inflammation that happens in some people with psoriasis. About one in five people with psoriasis will get arthritis, but it's not clear why some people get it and others don’t. More than half of the patients with arthritis get joint damage, which can limit their daily activities. The main goal of treating patients is to improve their quality of life, help them function normally, and keep them productive at work. Unfortunately, the burden of psoriatic disease is often significant and reduces the quality of life for those affected. Patient questionnaires help evaluate how the disease is responding to treatment, its impact on quality of life, and its effect on patients’ well-being. In this review, we looked at existing research on how PsA affects patients’ quality of life. We identified 37 studies that were relevant to patients with PsA. We found that people with PsA generally have a lower quality of life, especially in terms of their emotions, social interactions, and mental well-being. Those who reported severe pain and fatigue also reported lower quality of life scores. Moreover, if their disease was poorly controlled, it negatively affected work productivity and employment satisfaction scores. Additionally, they reported difficulties with sleep, low mood, and challenges with sexual function. This review summarised that psoriatic arthritis greatly impacts patients’ quality of life and daily activities. Therefore, we suggest that along with regular check-ups, it’s important to address patients’ emotional, social, and mental aspects with compassionate care.

## Introduction

Psoriatic arthritis (PsA) is a heterogeneous chronic inflammatory musculoskeletal condition that occurs in 30% of those with psoriasis (PsO). Across Europe, the prevalence of PsA is estimated at 207 per 100,000.^1^ Over half of patients with PsA have a progressive and erosive disease, resulting in functional impairment.^2,3^ PsA presents with diverse manifestations, including peripheral arthritis, enthesitis, dactylitis, spondylitis and skin/nail PsO, which makes each patient’s experience unique.^4^ The impact of PsA on patients’ lives is multifaceted and challenging to measure uniformly.

Previous studies have linked PsO and arthritis with a significant psychological burden, including depression, risky health behaviour and negative body image.^5^ Patient reported outcome (PRO) measures are an important component of assessing disease impact and therapy response in patients with PsA. A wide range of PROs exist; few have been developed or adapted specifically to PsA.^6,7^

The concept of health-related quality of life (HRQoL) has been defined by the International Society for Quality-of-Life Research as: ‘the functional effect of a medical condition and/or its consequent therapy upon a patient. Health-related quality of life is thus subjective and multidimensional, encompassing physical and occupational function, psychological state, social interaction and somatic sensation’.^8^ Various health instruments, when used consistently, can capture these data in clinical practice and clinical trials; these include the PsA Quality of Life Index and Euro-QoL 5 Dimensions. Similarly, PROs are used to assess pain, disease activity (joints and skin), disability and physical function, fatigue and productivity when assessing the health status of people with PsA.^9^

The objective of this review was to investigate/describe published research on the impact of PsA on patients’ QoL and physical function.

## Materials and methods

The review protocol was pre-registered with PROSPERO (registration number CRD42021257395) and followed through all stages of the review. This systematic review is reported in accordance with the Preferred Reporting Items for Systematic Reviews and Meta-Analyses (PRISMA) guidelines^10^ and the PRISMA for Abstracts checklist.^11^ A copy of the PRISMA checklist is presented in Supplemental File 1.

### Types of participants

Adults over the age of 18 who are affected by PsA with a diagnosis made by a Rheumatologist or who met validated classification criteria (e.g. ‘classification of psoriatic arthritis (CASPAR) criteria’).^12^ Studies with participants who have PsO or axial spondyloarthritis were only included if they reported outcomes for PsA separately or if separate data were available from study authors upon request. Studies were excluded if the participants were under the age of 18 or if they did not have psoriatic disease as their primary diagnosis.

### Types of outcome measures

The chosen PROs were selected in accordance with the suggestions of the OMERACT (Outcome Measures in Rheumatology) Groups PsA Core Domain Set.^13^ The GRADE (Grading of Recommendations, Assessment, Development and Evaluation) Working Group recommends the inclusion of studies that evaluate at least one primary outcome.^14^ The primary outcome measures were PROs reporting on QoL and impact of PsA on patients. The secondary outcome measures considered included fatigue, anxiety, depression, sleep and work productivity.

### Literature search

On the 28 May 2021, a systematic search was performed in the Cochrane Central Register of Controlled Trials (CENTRAL) (via Cochrane Library, Wiley), CINAHL (via EBSCOhost), AMED (via OVID), EMBASE (via OVID), Global Health (via OVID), MEDLINE (via OVID) and PsycINFO (via OVID) to retrieve articles related to QoL and lifestyle in adults with PsA.

The search strategy was designed to be broad to ensure retrieval of all relevant studies related to QoL or lifestyle issues in patients with PsA and utilized a PsA specific facet of a search strategy developed by the GRAPPA 2021 Treatment Recommendations guideline group.^15^

The search terms included relevant controlled vocabulary headings (e.g. MeSH, EMTREE) for each database and free-text terms (searched in the title, abstract or keyword fields) for ‘PsA’ and lifestyle or non-pharmacological or psychological search terms. A date limit of 2010–2021 was applied to the search but no other limits were used. A copy of the search strategies for all the databases searched can be found in Supplemental File 2.

The review team are conducting two similar reviews (see PROSPERO protocol 257395). Each review has a different focus, but the same search approach and strategies have been used to identify studies for both reviews.

### Study selection

Titles and abstracts were screened by two independent reviewers (L.H.H. and D.M.), who also assessed full texts for inclusion and performed data extraction from eligible studies. Conflict at any stage was resolved through discussion moderated by a third reviewer (R.A.).

### Data extraction and assessment of the risk of bias

A standardized data extraction sheet was developed within Covidence and piloted (L.H.H. and L.J.). Two independent reviewers (L.J. and R.S.) conducted data extraction and Risk of Bias analyses. Any disagreements were resolved by the third reviewer (L.H.H.). The methodological quality, validity and credibility, and the quality of the included studies was evaluated according to the Critical Appraisal Tool for Analytical Cross-Sectional Studies of the Joanna Briggs Institute as shown in Supplemental File 3.^16^ The following results were extracted from each paper: PROs on QoL and function, including disability index scores. Additionally, as secondary outcomes, PROs on fatigue, anxiety, depression, sleep, work productivity and employment status were also collected.

### Analysis and synthesis methods

We expected there to be considerable differences in the participants, interventions and outcomes among the studies included. We recorded the details of each study’s participants, interventions and outcomes. Since the continuous data did not include means and standard deviations, we could not conduct statistical analysis. The significant variations in the reported data prevented us from performing a meta-analysis. As a result, we used a narrative synthesis approach to analyse the results.

## Results

### Search results

**Figure 1.**
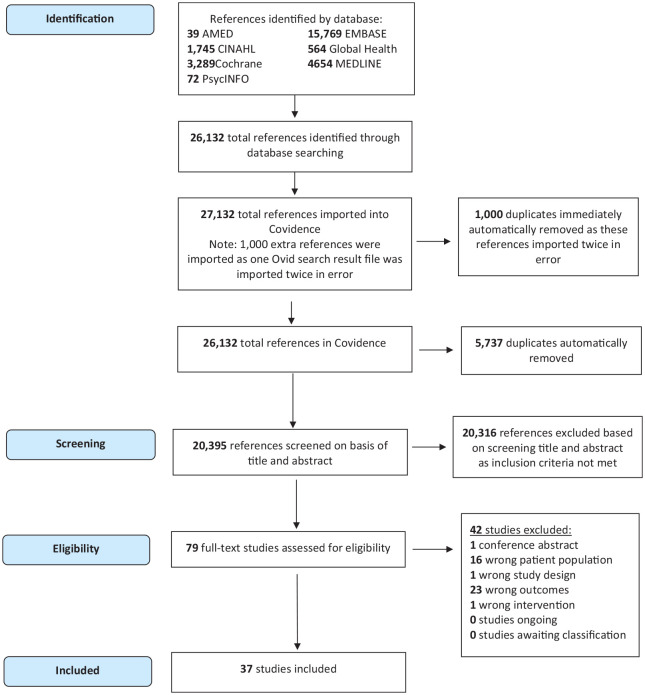
Preferred Reporting Items for Systematic Review and Meta-Analysis (PRISMA) flow diagram for study selection. AMED = Allied and Complementary Medicine Database; CENTRAL = Cochrane Central Register of Controlled Trials; CINAHL = Cumulative Index to Nursing and Allied Health Literature; EMBASE = Excerpta Medica DataBASE.

The literature search retrieved a combined total of 26,132 references. The references were imported into Covidence. An additional, 1000 references were imported into Covidence in error as one Ovid results file was imported twice. These additional 1000 references were immediately removed by the Covidence automatic deduplication function. The automatic deduplication also identified an additional 5737 duplicate references. The remaining 20,395 references then underwent title and abstract screening. Following initial screening, 79 articles were assessed for eligibility with a full-text review. Following this, 37 articles were included for narrative synthesis, as shown in the PRISMA flow diagram in Figure 1.

### Primary outcomes

The 37 studies included in the review are shown in Table 1. Out of the 37 included studies, 23 studies reported measures of HRQOL as shown in Table 2 and 24 reported measures of impact of PsA on function (Table 3).

**Table 1.** Final studies included in this review and the outcome measures.

Total number of studies (*n* = 37)	No. PsA participants	Quality of life scores (*n* = 23)	Impact of PsA scores (*n* = 24)
Ballegaard et al. (2020)	100	X	X
Bandinelli et al. (2013)	100	–	X
Baskan et al. (2016)	52	X	X
Baviere et al. (2020)	124	X	–
Billing et al. (2010)	123	X	–
Brihan et al. (2020)	54	X	–
Cano-Garcia et al. (2021)	149	X	–
Carneiro et al. (2017)	101	X	X
Chiowchanwisawakit et al. (2019)	129	X	X
Coates et al. (2020)	1286	–	–
Conaghan et al. (2020)	640	X	X
Dalal et al. (2015)	107	X	–
diMinno et al. (2013)	270	X	–
Duvetorp et al. (2019)	1264	–	X
Geijer et al. (2021)	166	–	X
Gezer et al. (2017)	41	X	–
Gratacos et al. (2014)	287	X	X
Gudu et al. (2016)	246	–	X
Haugeberg et al. (2020) – ID 13000	135	X	–
Haugeberg et al. (2020) – ID 13003	131	X	–
Haugeberg et al. (2020) – ID 13009	137	–	X
Howells et al. (2018)	179	X	–
Kotsis et al. (2012)	83	X	–
Krajewska-Wlodarczyk et al. (2018)	62	X	X
Kwan et al. (2019)	17	–	X
Lai et al. (2021)	231	–	X
Lindqvist et al. (2017)	58	X	X
Mease et al. (2017)	1240	–	X
Merola et al. (2019)	439	X	–
Mulder et al. (2022)	855	–	X
Orbai et al. (2020)	458	–	X
Palominos et al. (2020)	396	–	X
Rodrigues et al. (2019)	20	X	X
Tezel et al. (2015)	80	X	X
Tillett et al. (2015)	318	X	X
Wervers et al. (2019)	268	–	X
Wong et al. (2022)	743	–	X

PsA, psoriatic arthritis.

**Table 2.** Outcome measures related to quality of life.

Measure	Study name (*n* = no. of PsA participants)	No. of PsA participants	Subgroup	Results	Comparator	Comparator results	Significance	Measure
PsAQoL	Tezel 2015	80		6.85 ± 5.37	PsO Control	6.99 ± 5.88 3.29 ± 4.23		Mean ± SD
	Baskan 2016	52		6.90 ± 6.28				Mean ± SD
	Billing 2010	123	General health status: -Poor -Fair -Good -Very good	13.5 ± 4.6 7.3 ± 4 1.9 ± 2.3 0.6 ± 0.7	Flare-up type: -PsA + PsO -PsA only -PsO only -None (no flare)	9.1 ± 5.0 6.6 ± 5.1 3.5 ± 4.5 4.3 ± 4.4		Mean ± SD
	Gezer 2017	41		11.36 ± 6.09				Mean ± SD
	Howells 2018	179		13.0, 9.0				Median, IQR
	Merola 2019	439		9.1 ± 6.1				Mean ± SD
EQ-5D Health utility score (0–1.0) EQ VAS (range 0–100)	Tillett 2015	318		0.4 ± 0.36				Mean ± SD
	Rodrigues 2019	20		0.71 ± 0.25	SpA (*n* = 32)	0.66 ± 0.35		Mean ± SD
	Gratacos 2014	287		0.64 ± 0.25				Mean ± SD
	Chiowchanwisawakit 2019	129		0.76 ± 0.23 68.6 ± 20.2				Mean ± SD
EQ-3D	Conaghan 2020	640	Low fatigue (BP score 76–100)	0.756 ± 0.288			*p* < 0.0001 *p* < 0.0001	Mean ± SD
			Severe fatigue (BP score 0–52)	0 .790 ± 0.213				
			Low pain (VT score 66–100)	0.940 ± 0.100				
			Severe pain (VT score 0–50)	0.776 ± 0.247				
DLQI (0–30)	Tillett 2015	318	Not working, *n* = 92	5 ± 5.9				Mean ± SD
			Working, *n* = 236	4 ± 5.3				
	Merola 2019	439		9.7 ± 8.4				Mean ± SD
	Haugeberg 2020 – ID 13003	131		3.3 ± 3.6				Mean ± SD
	Haugeberg 2020 – ID 13000	135		3.4 ± 3.7				Mean ± SD
	Ballegaard 2020	100		1 (0.0, 4.5)	PsO (*n* = 20)	5 (2.0, 7.5)	*p* = 0.002	Median (range)
	Dalal 2015	107		3 (0, 9)	PsO (*n* = 145)	6 (3, 10)	*p* = 0.009	Median (range)
SF-36 Range (0–100) 0 indicating the lowest level of function and 100 the highest level of function	Cano-Garcia 2021	149	SF-36 PCS SF-36 MCS	32.9 ± 12.8) 49.1 (33.6–55.8	Axial SpA, (*n* = 152)	30.7 ± 15.8 48.5 (33.4–56.1)		Mean ± SD, Median (IQR)
	Carneiro 2017	101	Functional capacity Physical aspects limit. Pain General health status Social aspects Mental health Emotional aspect Vitality	59.52; 60 51.04; 50 52.59; 42 59.81; 62 63.72; 62.50 65.71; 68 66.77; 97 58.5; 60				Mean and median
	Bavière 2020	124	PCS -Physical function -Role limitations -Bodily pain General health perception MCS -Vitality -Social function -Role limitations -General mental health	41.2 ± 9.7, 40.8 62.3 ± 26.3, 70.0 48.4 ± 40.9, 50.0 48.9 ± 26.2, 51.0 47.2 ± 20.2, 46.0 43.2 ± 12.4, 47.0 43.6 ± 20.6, 45.0 70.1 ± 27.5, 75.0 59.4 ± 43.7, 66.7 58.7 ± 21.3, 60.0				Mean ± SD, median
SF-12 PCS Sf-12 MCS Range (0–100)	Dalal 2015	107		41.7 ± 11.6 49.6 ± 9.6	PsO, *n* = 145	49.3 ± 10.2 46.6 ± 12.2	*p* < 0.001 *p* = 0.04	
WHOQOL-BREF; -Physical -Mental -Social -Environment	Kotsis 2012	83		58.96 ± 2.44 61.09 ± 2.37 56.95 ± 2.89 60.28 ± 2.07	Rheumatoid arthritis, *n* = 199	58.62 ± 1.41 65.14 ± 1.35 60.27 ± 1.65 64.46 ± 1.18	NS NS NS NS	Mean ± SEM
PSQI	Gezer 2017	41	Global PSQI; PSQI ⩾6	9.70 ± 3.90; 85%	HC (*n* = 38)	4.05 ± 1.85; 29%	<0.001	Mean ± SD; %
	Krajewska-Wlodarczyk 2018	62	Global PSQI (0–21); PSQI ⩾6	9.32 ± 5.02 68%	PsO (*n* = 52) HC (*n* = 41)	7.11 ± 4.31 58%. 4.05 ± 2.91; 14%	*p* = 0.027 PsO vs PsA global PSQI	Mean ± SD; %

BP, bodily pain; DLQI, Dermatology Life Quality Index; EQ-5D, EuroQoL 5-dimensional questionnaire; HC, healthy control; IQR, interquartile range; MCS, mental component summary; PCS, physical component summary; PsAQoL, Psoriatic Arthritis Quality of Life; PsO, psoriasis; PSQI, Pittsburgh Sleep Quality Index; SF-36, 36-Item Short Form Survey; SpA, Spondyloarthritis; VAS, visual analogue scale; VT, vitality; WHOQOL-BREF, World Health Organization Quality of Life Instrument, Short Form.

**Table 3.** Outcome measures related to impact of PsA and function.

Outcome measurement	Study name	No. of PsA participants	Subgroups	Results	Comparator	Comparator results	Significance	Measure
PsAID	Orbai 2020	458	Overall Women (*n* = 228) Men (*n* = 230)	3.4 ± 2.5 4.1 ± 2.4 2.8 ± 2.3				Mean ± SD
	Ballegaard 2020	181		5.2 (3.0–6.5)				Median (IQR)
MAPP survey Question: ‘Does diagnosis impact on activities of daily living’ (number)	Duvetorp 2019	1264	PsA + PsO	None: 27% 1–3: 28.6% 4+: 44.5%	PsO	None: 61.9% 1–3: 22.7 4+: 15.4		%
RSE	Brihan 2020	54	PsA/severe PSO (*n* = 54)	24.33 ± 6.24	Mild PsO, *n* = 56	26.53 ± 3.40	Significant *p* = 0.001	Mean ± SD
HAQ	Lindqvist 2017	58		0.56 ± 0.45	PsA mutilans *n* = 67	0.98 ± 0.81	*p* = 0.001	Mean ± SD
	Mease 2017	1240	Total	0.63 ± 0.65				Mean ± SD
			BSA ⩽3%, (*n* = 789)	0.56 ± 0.61				
			BSA >3%, (*n* = 451)	0.76 ± 0.71				
	Gratacos 2014	287		0.76 ± 0.67				Mean ± SD
	Krajewska-Wlodarczyk 2018	62		0.89 ± 0.5				Mean ± SD
	Rodrigues 2019	20		0.48 ± 0.75	SpA (*n* = 32)	0.56 ± 0.95		Mean ± SD
	Wong 2022	743		0.5 ± 0.6				Mean ± SD
	Gudu 2016	246	Total score	0.99 ± 0.72				Mean ± SD
			High fatigue (n = 110)	1.37 ± 0.60				
			Low fatigue (*n* = 136)	0.68 ± 0.66				
	Tillett 2015	318	Working	1.3 ± 0.78				Mean ± SD
			Not working	1.0 ± 0.68				
	Mulder 2022	855	Men	0.39 ± 0.5				Mean ± SD
			Women	0.87 ± 0.7				
	Haugeberg 2020 – ID 13009	137		0.44 ± 0.40				Mean ± SD
	Geijer 2021	166		0.59 ± 0.53	RA (*n* = 131)	0.85 ± 0.58	*p* = 0.011	Mean ± SD
	Conaghan 2020	640		0.6 ± 0.7				Mean ± SD
	Orbai 2020	458	Overall	0.7 ± 0.7				Mean ± SD
			Women (228)	0.9 ± 0.7				
			Men (230)	0.5 ± 0.6				
	Carneiro 2017	101		0.85 (0–2.9), 0.88				Mean (range), median
	Chiowchanwisawakit 2019	129		0.38 (0.1, 1.1)				Median (range)
	Wervers 2019	94	Early MDA	0.13 (0.00–0.63)				Median (IQR)
		77	Late MDA	0.63 (0.38–0.89)				
		97	Never MDA	0.88 (0.63–1.38)				
	Ballegaard 2020	181		0.88 (0.38–1.25)	PsO Healthy controls	0 (0–0.32) 2.0 (0.0–4.0)	*p* < 0.001	Median (IQR)
	Dalal 2015	107		0.4 (0, 0.9)	PsO (*n* = 145)	0 (0, 0.1)	*p* < 0.001	Median (range)
	Lai 2021	231		0.13 (0–0.63)				Median (range)
	Kwan 2019	17		0.125 (0–1.625)				Median (range)
HAQ (mild: 0 to ⩽1, moderate: >1 to 2, severe: >2 to 3)	Palominos 2020	396		Mild: 74% moderate: 22.5% severe: 3.5%				%
HAQ-S (for the spondyloarthropathies)	Tezel 2015	80		0.49 ± 0.5	PsO (*n* = 40) Control (*n* = 40)	0.23 ± 0.46 0.12 ± 0.26	*p* < 0.05 (PsA and PsO)	Mean ± SD
	Mease 2017	1240	Total (*n* = 1240)	0.63 ± 0.65				Mean ± SD
			BSA ⩽ 3%, (*n* = 789)	0.56 ± 0.61				
			BSA >3%, (*n* = 451)	0.76 ± 0.71				
	Baskan 2016	52		0.50 ± 0.50				Mean ± SD
BASFI	Bandinelli 2013	100		1.7 ± 2.1 >0: 81%				Mean ± SD %

BASFI, Bath Ankylosing Spondylitis Functional Index; BSA, body surface area; HAQ-S, Health Assessment Questionnaire for Spondyloarthropathies; IQR, interquartile range; MAPP, Multinational Assessment of Psoriasis and Psoriatic Arthritis; MDA, minimal disease activity; PsA, psoriatic arthritis; PsAID, psoriatic arthritis impact of disease; PsO, psoriasis; RA, Rheumatoid arthritis; RSE, Rosenberg Self-Esteem Scale.

### Quality of life outcome measures

#### Psoriatic arthritis quality of life

Psoriatic arthritis quality of life (PsAQoL) is a disease-specific measure of QoL^17^ with 20 questions with binary true/false scoring. The overall score can range from 0 to 20, a high score indicates poor QoL.^17–19^ In this systematic literature review, six studies,^20–25^ looked at PsAQoL. The total number of participants with PsA was 914 (range *n* = 41–439), combining all six studies. Participants had a mean age range between 41.4 and 49.4, and mean disease duration ranged from 24 to 58 months. The mean PsAQoL score was reported in five out of the six studies, ranging from 5.8 ± 5.2 to 13.5 ± 4.6, and a median score of 13.0 in the remaining study. These studies have examined various factors contributing to this finding, such as the impact on work-related QoL,^20^ high rates of anxiety and depression^24^ and impaired sleep quality,^23^ both factors were associated with reduced PsAQoL.

Billing et al.^22^ used PsAQoL scores by self-reported general health and flare-up status in the Swedish population. There were 123 patients 28 with early PsA (diagnosed less than 2 years), 75 with PsA for over 2 years and 20 did not specify duration, with a mean PsA duration of5 ± 3.9 (1–25) in years and mean PsO duration of 14.7 ± 12.6 (1–69). Those who reported poor general health had the highest mean PsAQoL (13.5 ± 4.6 vs 0.6 ± 0.7). Similarly, the presence of PsO and arthritic symptoms simultaneously had a significantly higher mean score (9.1 ± 5.0; worse QoL) than either symptom independently or with no flare (mean score of 4.3 ± 4.4). As this was a study to evaluate the reliability, psychometric and acceptability standards of the PsAQoL questionnaire, disease severity was not reported.

#### EuroQoL 5-dimensional questionnaire

The EuroQoL 5-dimensional questionnaire (EQ-5D) is a reliable and simple tool for measuring health outcomes across diseases. It assesses five dimensions of health, including mobility, self-care, usual activities, pain and discomfort, and anxiety and depression.^26^ The EQ-5D-3L and EQ-5D-5L versions featuring three and five response levels for each dimension, respectively. A health utility score is a single utility score that reflects an individual’s health state at a particular point in time (ranging from 0 to 1.0); a number close to 1.0 reflects good health. The overall health using a visual analogue scale (VAS) is also measured.^26–28^

The EQ-5D-5L was measured in five studies,^29–32^ whereas the EQ-5D-3L version was used in one study.^33^ The combined number of participants was 754, with an average age range of 47.6–52.6 and a range of disease duration between 96 and 123 months. Out of all the studies, a UK-based study had the lowest mean health utility score of 0.4^29^ (*n* = 318). The mean health utility score ranged from 0.4 ± 0.36 to 0.76 ± 0.23.^32^ Two studies also measured EQ-VAS, and the average was 64.5. Conaghan et al.^33^ found that as levels of pain and/or fatigue escalated from low to moderate to severe, a notably greater proportion of patients indicated experiencing ‘some’ or ‘extreme’ difficulties with mobility, self-care, usual activities, pain and anxiety/depression according to the EQ-5D-3L measurement.

#### Dermatology Life Quality Index

Dermatology Life Quality Index (DLQI) is a valuable tool used to assess the impact of skin diseases on an individual’s QoL.^34^ It is a validated 10-item questionnaire (each item scoring 0–3) assessing the impact that any dermatological condition over the past week.^34,35^ Scores range from 0 to 30^36^ in categorical variable: 0–1 (none), 2–5 (small), 6–10 (moderate), 11–20 (very large) and 21–30 (extremely large) demonstrating the impact on QoL.

In total, six studies have used DLQI to measure QoL in PsA.^29,25,37–40^ There was a total of 1230 participants in the studies, with a mean age range of 48.0–52.1. Out of the six studies, the percentage of females who participated ranged from 48% to 52%. Meanwhile, four studies reported that the mean duration of disease ranged from 8 to 22.2 years. The mean DLQI was reported in four out of the six studies, ranged from 3.3 ± 3.6 to 9.7 ± 8.4. The study with the largest number of participants, Merola et al.^25^ (*n* = 439, PsA with PsO), revealed that poorly controlled skin disease had a significant impact on patient QoL (mean DLQI 9.7 ± 8.4). Multivariate analyses showed that severity of joint symptoms was associated with lower QoL (*t* = 13.15), followed by impact of skin symptoms (*t* = 5.11). The median DLQI was reported in two of the studies.^39,40^ Both studies compared differences in QoL between patients with PsO alone and those with PsA. Compared to individuals with PsA, individuals with PsO alone had a higher DLQI.^39^

#### 36-Item Short Form Survey

36-Item Short Form Survey (SF-36) is a very popular measure of HRQoL across diseases. SF-36 is a comprehensive questionnaire (36 questions) that assesses eight health concepts and subscales, with zero representing maximum health impairment and 100 no health impairment.^41^ The total score comprises a physical component summary (PCS) and mental component summary (MCS) measures (range: 0–100). SF-12 is a shorter version of the SF-36 questionnaire, designed to capture essential aspects of HRQoL.^42^ It consists of 12 questions that assess both physical and mental health components.^42^ In total, three studies evaluated QoL using SF-36.^43–45^ There were 374 patients combined, mean age ranged from 49.4 to 52.6, and mean duration of disease ranged from 8.2 to 11.3 years.

A study by Bavière et al.^43^ showed that within the physical component, the PsA group (*n* = 124) demonstrated the lowest scores in role limitation due to physical health problems and bodily pain sub-domains. They also exhibited the lowest scores in vitality and general mental health within the mental component. The study delved deeper into the link between comorbidities and QoL in PsA by employing the modified rheumatic disease comorbidity index. The study also found that anxiety in PsA was independently associated with QoL, and specifically with mental health. The second study by Cano-Garcia et al.^44^ assessed the impact of insomnia on QoL in PsA patients (*n* = 149). The overall score in physical and mental health components was low in the PsA group, with the mean score of PCS being 32.9 ± 12.8 and the median score for MCS being 49.1 (33.6–55.8). Multivariate regression analysis showed that insomnia was inversely associated with emotional recovery and directly associated with depression in axial spondyloarthritis (AxSpA) and PsA. However, the study failed to show any significant association between insomnia and QoL in PsA patients. This study had two groups of patients with AxSpA and PsA, and combining the conditions limits conclusions for PsA.

Carneiro et al.^45^ assessed the prevalence of fatigue and correlated it with QoL indexes. The study measured the mean and median scores of all subdomains within the SF-36 in PsA patients (*n* = 101) and found that disease impaired all SF-36 domains. The multivariate analyses showed a strong correlation between Functional Assessment of Chronic Illness Therapy – Fatigue (FACIT-F) and the different domains of SF-36 including physical and functional aspects and emotional, social and mental health.

All three studies have several limitations that should be considered. The cross-sectional design, for instance, prevents us from establishing causal associations between the findings, indicating the need for longitudinal studies. Additionally, the multicentre nature of the studies introduces a certain degree of variability in the care provided, suggesting the need for standardized protocols. Lastly, all three studies employed disease activity scores that were nonspecific to PsA, such as DAS-28, BASDAI and clinical disease activity index score, highlighting the need for greater specificity with disease-specific measures in future research.

In a univariate cross-sectional analysis using the MCS and PCS components of the SF-12 questionnaire, Dalal et al.^40^ demonstrated that PsA diagnosis was associated with a lower mean SF-12 PCS score (41.7 ± 11.6 vs 49.3 ± 10.2, *p* value < 0.001) and a higher mean SF-12 MCS score (49.6 ± 9.6, vs 46.6 ± 12.2, *p* value = 0.04) when compared to patients with PsO only. On multivariable models, after adjusting for factors such as age, gender and body mass index, the association between PsA and PsO on the MCS score was attenuated. However, the association between PsA diagnosis and lower PCS score remained. Despite limitations, the studies highlight that PsA patients experience a range of physical functions, and that the disease can have a variable impact on their mental, emotional and social well-being.

#### World Health Organization Quality of Life Instrument, Short Form

The World Health Organization Quality of Life Instrument, Short Form (WHOQOL-BREF) is a 26-item QoL instrument divided into 4 domains: physical health (7 items), psychological health (6 items), social relationships (3 items) and environmental health (8 items). Each individual item is evaluated using a 5-point Likert scale ranging from 1 to 5. The domain scores are interpreted in a positive light, meaning that lower scores indicate lower QoL. The domain score is calculated by taking the average score of all items within that domain, which is then linearly transformed to a scale ranging from 0 to 100.^46,47^

One study^48^ using WHOQOL-BREF found that the psychological factors specifically are associated with lower overall HRQOL in PsA. The authors used a wide range of mental health (Patient Health Questionnaire 9, PHQ-9) and illness beliefs (Brief Illness Perception Questionnaire) to explore the mediating effect of these factors on HRQoL in PsA. They found that after controlling for disease duration and pain level, anxiety symptoms as measured on PHQ-9 and ‘concern about bodily symptoms attributed to condition’ contributed to a statistically significant lower HRQoL score. This study is limited by the cross-sectional design and exclusion of pain scores.

### Impact of disease outcome measures

#### Psoriatic arthritis impact of disease-12

Psoriatic arthritis impact of disease-12 (PsAID-12) is a 12-item questionnaire with a scale range from 0 to 10.^49^ It captures domains of pain, fatigue, skin problems, work and/or leisure activities, functional capacity, discomfort, sleep disturbance, coping, anxiety, embarrassment and/or shame, social participation and depression.^50^ A higher PsAID-12 score indicates poorer patient-reported status.^50^ In this cross-sectional study,^51^ having a PsAID score ⩾4 (high life impact) was associated with female sex, enthesitis, tender joints and comorbidities; and independently associated with female sex. Ballegaard et al.^39^ aimed to explore the prognostic value of pre-specified comorbidities on treatment outcomes in PsA. Additionally, the authors compared baseline data with two control populations: patients with cutaneous PsO without arthritis, and healthy controls (HCs). Obesity, hypertension and the presence of one or more conditions on Charlson Comorbidity Index were each independently associated with poorer treatment outcome rates in PsA. Patients with any comorbidities had significantly lower PsAID-12 scores at follow-up. Additionally, both pain and fatigue were prominent subdomains of PsAID that significantly affected patients’ HRQoL.

#### Multinational Assessment of Psoriasis and Psoriatic Arthritis survey

A study conducted by Duvetorp et al.^52^ examined the effects of psoriatic disease on QoL in patients in Sweden, Denmark and Norway. The researchers used the Multinational Assessment of Psoriasis and Psoriatic Arthritis (MAPP) survey to invite 1264 individuals who had reported a physician’s diagnosis of PsO/PsA. The majority of patients with PsA ± PsO (73%) reported at least one impact of the disease, and 44.5% reported four or more impacts on their daily activities. More than half of the participants (52%) with PsA and PsO reported missing work or school, whereas for those with PsO alone, the figure was 15%. However, 62% of participants with PsO alone stated that their disease did not significantly impact their daily activities.

#### Rosenberg Self-Esteem Scale

Rosenberg Self-Esteem Scale (RSES) is a validated instrument for measuring changes in self-esteem.^53^ The test comprises of 10 statements that assesses an individual’s self-worth or self-acceptance.^54^ Each statement is assigned a score value ranging from 0 to 3, with a higher score indicating higher self-esteem.^54^

RSES was reported in one study by Brihan et al.,^55^ which included 54 patients with PsA, with an age range of 26–74 years and 38% being female. The study examined the impact of PsO on the self-esteem of 110 patients, divided into severe cutaneous PsO and PsA and patients with mild PsO-only groups. The study found that patients with severe cutaneous PsO and PsA had lower self-esteem than those with mild PsO alone. The statistical analysis showed a lower self-esteem in PsA + PsO group compared with the PsO alone, *t*(81.34) = −2.286 significant at a *p* = 0.025 threshold (*m*_psoriasis arthritis_ = 24.33 and *m*_mild psoriasis_ form = 26.53, for a *F* (FLevene’s) = 21.765; *p* < 0.001, *p* < 0.05). The study did not delve into specific comorbidities or their effects on individuals with PsO or PsA.^56^

#### Health Assessment Questionnaire

Health Assessment Questionnaire (HAQ) is a measurement of physical function in PsA.^57^ The HAQ Disability Index is scored as: ‘mild to moderate difficulty’ (score <0.8); ‘moderate to severe disability’ (0.8 ⩽ score < 1.2) and ‘severe to very severe disability’ (score ⩾ 1.2).^42^

In this systematic literatire review, 20 studies^29–33,39,40,45,51,58–68^ measured HAQ score in PsA patients. The total number of participants with PsA was 6183 (range *n* = 17–1240), combining all 20 studies. The overall age range of participants extended from 46 to 56 years, and average disease duration ranged from 8 months to 28 years. The mean HAQ score was reported in 14 out of the 20 studies, ranging from 0.39 ± 0.5 to 1.3 ± 0.78. The remaining six studies reported median score, ranging from 0.12 to 0.88, which indicates mild-to-moderate difficulty.

Mulder et al.^63^ assessed differences between men and women in disease activity/HRQoL parameters and showed worse HAQ scores in women compared to men. Women also had higher disease burden, as defined by the PsA Disease Activity Score. Gudu et al.^62^ found that participants experiencing high fatigue (*n* = 110; fatigue numeric rating scale (NRS) score >5) reported the highest mean HAQ score 1.37 ± 0.60, a score >1.2 being ‘severe to very severe’ disability. Similarly, Tillett et al.^29^ explored the differences in HAQ score between working and non-working group of people with PsA, with a mean duration of disease for 8 years. The working group (*n* = 236) reported an average HAQ score of 1.0 ± 0.68, compared to HAQ score of 1.3 ± 0.78 in non-working group (*n* = 96). Moreover, this study also highlighted that older age, recent disease onset (2–5 years’ disease duration) and worse physical function exert a negative influence on remaining in employment. This cohort study also indicated that joint disease exerts the greatest influence on work disability.

Palominos et al.^69^ (*n* = 396) measured HAQ in PsA participants with and without sleep impairment. The mean age 51.9 ± 12.6 years, and 51% were female participants. In total, majority (74%) had mild disability, 22.5% reported moderate disability and 3.5% had severe disability score.

The HAQ for Spondyloarthropathies (HAQ-S) was formed with the addition of questions that evaluate the functions of the cervical and lumber spine. Daltroy et al.^70^ developed the HAQ-S by adding five questions that evaluate spinal mobility to the HAQ in 1990. HAQ-S score was reported in three studies,^20,21,59^ a total of 1372 participants with mean age between 42 and 54 year and 4.8 and 12.7 years of duration of disease. The mean (SD) HAQ-S score range from 0.49 ± 0.35 to 0.63 ± 0.65. Tezel et al.^20^ had two comparators in addition to PsA (*n* = 80): PsO (*n* = 40) and HC (*n* = 40). The HAQ was comparably higher in the PsA group, and the difference observed between the PsA and PsO groups was significantly different (*p* < 0.05), even after adjusting for other relevant factors. Substantial skin involvement (BSA >3%) is associated with a more significant PsA disease burden and a higher HAQ. The HAQ-S data suggests that physical functional status in the PsA group was worse than PsO, and physical pain and disability were the most significant factors affecting QoL.

#### Bath Ankylosing Spondylitis Functional Index

Bath Ankylosing Spondylitis Functional Index (BASFI) score evaluates functional limitation in patients with the inflammatory autoimmune disease ankylosing spondylitis (AS) based on a VAS.^71^ Sacroiliitis and/or spondylitis affects approximately 20%–40% of patients with PsA. BASFI comprises 10 tasks that assess the degree of difficulty of each task performed.

In a study of 100 participants^72^ with early PsA (symptoms duration less than a year), BASFI was used to evaluate the outcome measure. Although there was a comparator group with late PsA (disease duration over 10 years), the BASFI score was not provided for this group. The early group of PsA had a mean score of 1.7 ± 2.1 on the BASFI (normal range of BASFI being 0–10).

### Secondary outcomes

Secondary outcome included PROs on fatigue, anxiety and depression, sleep, work productivity and employment, intimacy, sexual life and emotional well-being (Table 4).

**Table 4.** Secondary outcome measures.

Outcome measurement	No. of studies	Results
Fatigue visual analogue scale (VAS) (mean ± SD)	5	47.8 ± 38 45.1 ± 32.4 45.5 ± 32.7 40.5 ± 29.3 60 (40–80), median (range)
Functional Assessment of Chronic Illness Therapy – Fatigue (FACIT-F) (mean ± SD)	5	22 ± 10.9 27.7 ± 8.7 37.5 ± 9.1 38.3 (mean) 23.0 (16.0–35.5) median IQR
Fatigue NRS (0–10) mean ± SD	5	7.9 ± 7.5 5.0 ± 3.0 4.2 ± 2.6 5.2 ± 3 4 (median)
Hospital Anxiety and Depression Scale (HADS) HAD A (mean)	4	7.7; 7.39; 9.73; 9.08
Hospital Anxiety and Depression Scale (HAD) HAD-D (mean)	4	6.5; 5.93; 9.09; 8.14
Patient Health Questionnaire 9 (PHQ-9)	1	PHQ-9 score >10 21.7% in PsA patients, 25.1% in RA patients and 36.7% in those PsA patients with polyarthritis.
Work productivity (WPAI) Mean (95% confidence interval) mean ± SD Percentage (%)	3	Total productivity at work = hours per week – absence – productivity loss at work, calculated in mean per week over past 4 week: Early MDA 32 (29–35), Late MDA 29 (25–34), never MDA 19 (14–25) Work productivity loss in the past 4 weeks was reported most often by never MDA patients 71% vs early MDA 27% vs late MDA 30%.
		Absenteeism 10.39 ± 19.81 Presenteeism 33.88 ± 28.98, Work productivity loss 37.15 ± 31.81 Activity impairment 43.78 ± 29.09
		Absenteeism 14%, Presenteeism 39%, Work productivity loss 40% Work activity impairment 49%.
Employment status	6	Employed (44%, 55%, 59%) vs Unemployed (56%, 1.4%, 6%, 16%). Full time employment (42%, 50%, 51%)
Intimacy, sexual life and emotional well-being	3	Emotional distress 58% Stopping social activities 45% Stopping sports/recreational activities 56% Social shame or disapproval 32%**Moderate/Major impact on:**Emotional/mental wellbeing 69%Romantic relationships/intimacy 56%Relationships with family and friends 44%Prevent wearing specific clothes/shoes 41.8% (PsA+PsO group)Present doing sports or leisure activity 44% (PsA+PsO group)Prevent participating in social activity 35.7% (PsA+PsO group)Prevent from an active sex life 28.8% (PsA+PsO group)Relationship difficulty with new friends/colleagues 24.3% (PsA+PsO group)Relationship difficulty with partner, close friends, relatives 22.1% (PsA+PsO group)

FACIT-F, Functional Assessment of Chronic Illness Therapy – Fatigue; Fatigue NRS, Fatigue numeric rating scale; HADS, Hospital Anxiety and Depression Scale; IQR, interquartile range; MDA, minimal disease activity; PHQ-9, Patient Health Questionnaire 9; PsA, psoriatic arthritis; RA, rheumatoid arthritis; VAS, visual analogue scale; WPAI, Work Productivity and Activity Impairment.

### Fatigue

The FACIT-F is a 40-item measure that assesses self-reported fatigue and its impact upon daily activities and function.^73^ Lai et al.^67^ reported mean FACIT-F score in 231 PsA participants, which was 37.5 ± 9.1. Of these, 22% reported severe fatigue, defined as FACIT-F score <30.^39^ PsA patients reported higher pain and fatigue scores, as well as more widespread pain compared to the control groups. In the study by Carneiro et al.,^45^ fatigue assessed by the FACIT-FS statistically significant correlated with the indices of QoL including HAQ score, HAD A and HAD D scores.

### Anxiety and depression

The Hospital Anxiety and Depression Scale (HADS) is used to measure anxiety (7 items) and depression (7 items).^74^ A score ⩾8 indicates a probable case of depression or anxiety.^75^

Kotsis et al.’s^48^ study (*n* = 83) investigated that the prevalence of moderate to severe levels of depressive symptoms (PHQ-9 score >10) was 21.7% in PsA patients, 25.1% in RA patients and 36.7% in those PsA patients with polyarthritis. After adjustment for severity of disease and pain, anxiety and concern about bodily symptoms attributed to the illness were independent correlates of physical HRQoL in PsA. The HADS score was collected in three studies,^31,45,72^ all three studies showed mild anxiety in PsA participants. However, only one study showed abnormal score in the depression scale.

### Sleep

The Pittsburgh sleep quality index (PSQI) is a questionnaire that assesses sleep quality and disturbances over a period of 1 month, based on patient-reported information.^76^ It uses a score between 0 and 21 to evaluate sleep quality, with a score of 6 or higher indicating poor quality.^76^

The first study, conducted by Krajewska-Wlodarczyk et al.,^60^ compared the mean global PSQI score (ranging from0 to 21) and the percentage of people with a PSQI score of 6 or higher (indicating poor sleep quality) between PsA (*n* = 62), PsO (*n* = 52) and HC groups (*n* = 41). The study found poor sleep quality in 67.7% of PsA patients, 57.7% in PsO patients and 14.6% within the control group. Sleep disorders in patients with PsA and PsO were related to worse QoL and intense fatigue. In a linear regression model, the following factors were found to worsen sleep quality in PsA: pain (*R*^2^ = 0.462, *p* < 0.001), tender joint count (*R*^2^ = 0.434, *p* < 0.001), C-reactive protein concentration (*R*^2^ = 0.391, *p* < 0.001), patient’s age (*R*^2^ = 0.284, *p* = 0.003) and duration of PsO (*R*^2^ = 0.166, *p* = 0.006).

In the second study, conducted by Gezer et al.,^23^ sleep disorders were observed in more than 85% of PsA participants (*n* = 41) who had diminished sleep quality, as defined by a PSQI score of 6 or higher. This was in contrast to only 29% of HC individuals (*n* = 38) who had poor sleep quality.

### Work productivity and employment

Work disability is an important functional outcome for PsA patients.^50^ The Work Productivity and Activity Impairment (WPAI)^77^ is a validated questionnaire that includes four domains. These domains are absenteeism, presenteeism, overall work productivity loss (which estimates both absenteeism and presenteeism) and impairment of non-work daily activities (which measures the percentage of daily activities affected by health issues) over the past week. Merola et al.^25^ (*n* = 439) found that joint severity and impact of arthritis were the strongest predictors of WPAI.

Tillett et al.,^29^ a large multicentre study, reported WPAI in working PsA group (*n* = 239); absenteeism 14%, presenteeism 39%, productivity loss 40% and activity impairment 49%.

A further 26% of participants were unemployed. Greater age, recent onset of disease and worse physical function were associated with higher risk of unemployment, whereas patient-reported employer helpfulness exerted a strongly positive influence on patients remaining in employment.

### Intimacy, sexual life and emotional well-being

The international global survey study by Coates et al.^49^ reported results from 1286 patients from 8 countries, showed that PsA had a moderate or major impact on emotional/mental well-being and 56% on romantic relationships/intimacy. Social impacts included emotional distress (58%), social shame or disapproval (32%) and ceased participation in social activities (45%). Comparing patients with PsO alone with those with PsO and PsA, Duvetorp et al.^52^ found that individuals with PsO alone, the most commonly reported strong negative impact was on their choice of clothing or shoes. For those with PsA and PsO, over 40% of respondents reported substantial negative impacts on their daily routine, leisure/sports and limitations on dress.

Assessment of QoL domains relating to social relationships are important for participants with PsA, as highlighted by Kwan et al.^68^ The relevance of social relationships, including sexual activity, was emphasized by 66.7% of patients with PsA through the use of the WHOQOL instrument and focus group discussions.

## Discussion

PsA can significantly impact various aspects of a patient’s life, including physical function, emotional well-being and social interactions. Pain, joint stiffness, fatigue and skin lesions can lead to decreased mobility, discomfort and limitations in daily activities. Joint symptoms can impact physical function and emotional health. Patients with more severe PsA tend to experience a greater impairment in HRQoL, whereas those with skin disease may have a negative impact on self-esteem and overall well-being. PsA-related fatigue can be overwhelming and may impact work performance and absenteeism. In particular, fatigue and pain can negatively affect work productivity. It is advisable to consider specific management plans that involve input from a multidisciplinary team to address these issues when present. This can help in maintaining the mobility and functional abilities of the patients, especially those with high disease activity.

This review summarizes data on the patient perspective in PsA using PROs. These outcomes allow us to evaluate a patient’s perception of their health status, including symptoms, function and other aspects of their life that may be impacted by the disease. Many of the measures used in PsA were initially developed for other diseases, such as HAQ DI for rheumatoid arthritis and FACIT-Fatigue for cancer-related anaemia.^6,78^ Some measures are generic and intended to evaluate overall population health status, including the SF-36 and the EQ5D.^6,79,80^ The PsAQoL index and PsAID are PsA-specific measures^17,49,81^ that were specifically developed for this patient population.^82^ Therefore, the adverse effect on QoL indicated in this review is likely to be underestimated. The studies conducted on the relationship between PsA, disease activity and sleep disorders are often restricted in their ability to draw conclusive results. Nevertheless, they draw attention to the impact that these disorders can have on patients, which is a crucial aspect of their QoL that is frequently overlooked in clinical practice.

The overall strengths of this review include the wide range of databases searched and the wide range of parameters considered for the overall impact of disease (physical symptoms (musculoskeletal and cutaneous), emotional and psychological, fatigues (both mental and physical), coping and sleep disturbance).

We identified five different PROs in the HRQoL category and five different PROs in impact of disease category. To comprehend the interrelation between HRQoL, disease impact and disease activity in PsA, it is necessary to adopt a comprehensive approach utilizing advanced statistical methods such as multilevel modelling. This is especially important considering the diverse nature of this disease course. However, despite a comprehensive review, there remains a lack of high-quality longitudinal studies on this topic, which leaves a significant question unanswered regarding the interconnectedness of all the factors. For example, the MAPP survey was a large international study; surveys offer valuable information about PsA and PsO. However, the survey lacked a control group and did not account for ethnic and healthcare system differences across countries. It was also limited by factors associated with methodology and enrolment requirements, including accurate recall and interpretation of questions, which may introduce recall bias or misclassification.

Pooling and comparing data for this review has been challenging due to the inconsistent use of various measures to capture HRQoL in cross-sectional settings, as well issues around data reporting. Additionally, the study relied on self-reported physician-diagnosed PsO/PsA, which may introduce recall bias or misclassification. Therefore, we have endeavoured to provide a narrative summary of the existing literature.

Since our review in 2021, multiple recent studies have been published, corroborating our findings.^83–85^ The studies concluded that psoriatic disease significantly impacts functional impairment, work productivity and QoL.

## Conclusion

This review highlights the significance of assessing PROs on QoL and the patient’s viewpoint on disease impact. This highlights the importance of integrating these insights into shared decision-making between patients and healthcare professionals. The impact of PSA on an individual’s HRQoL can vary greatly and have a substantial impact on their overall well-being.

## Supplemental Material

sj-docx-1-tab-10.1177_1759720X241295920 – Supplemental material for The impact of psoriatic arthritis on quality of life: a systematic review Supplemental material, sj-docx-1-tab-10.1177_1759720X241295920 for The impact of psoriatic arthritis on quality of life: a systematic review by Lija James, Louise H. Hailey, Rhea Suribhatla, Dylan McGagh, Raj Amarnani, Christine E. Bundy, Shona Kirtley, Denis O�Sullivan, Ingrid Steinkoenig, Jonathan P. E. White, Arani Vivekanantham and Laura C. Coates in Therapeutic Advances in Musculoskeletal Disease

sj-docx-2-tab-10.1177_1759720X241295920 – Supplemental material for The impact of psoriatic arthritis on quality of life: a systematic review Supplemental material, sj-docx-2-tab-10.1177_1759720X241295920 for The impact of psoriatic arthritis on quality of life: a systematic review by Lija James, Louise H. Hailey, Rhea Suribhatla, Dylan McGagh, Raj Amarnani, Christine E. Bundy, Shona Kirtley, Denis O�Sullivan, Ingrid Steinkoenig, Jonathan P. E. White, Arani Vivekanantham and Laura C. Coates in Therapeutic Advances in Musculoskeletal Disease

sj-docx-3-tab-10.1177_1759720X241295920 – Supplemental material for The impact of psoriatic arthritis on quality of life: a systematic review Supplemental material, sj-docx-3-tab-10.1177_1759720X241295920 for The impact of psoriatic arthritis on quality of life: a systematic review by Lija James, Louise H. Hailey, Rhea Suribhatla, Dylan McGagh, Raj Amarnani, Christine E. Bundy, Shona Kirtley, Denis O�Sullivan, Ingrid Steinkoenig, Jonathan P. E. White, Arani Vivekanantham and Laura C. Coates in Therapeutic Advances in Musculoskeletal Disease

## Declarations

**Ethics approval and consent to participate:** Ethical approval was not required. Consent to participate: Not applicable.

**Consent for publication:** There are no human participants in this article and informed consent is not required.

**Availability of data and materials:** All data generated or analysed during this study are included in this published article.
