# Supplementary material for: The impact of psoriatic arthritis on quality of life: a systematic review
Source: Ther Adv Musculoskelet Dis. 2024 Dec 22;16:1759720X241295920. doi: 10.1177/1759720X241295920 (PMC11664531; doi:10.1177/1759720X241295920)
Supplement: sj-docx-2-tab-10.1177_1759720X241295920 – Supplemental material for The impact of psoriatic arthritis on quality of life: a systematic review [file sj-docx-2-tab-10.1177_1759720X241295920.docx]

**Search strategies**

Literature search

The literature search was conducted on the 28 May 2021 in the Cochrane Central Register of Controlled Trials (CENTRAL) (via Cochrane Library, Wiley), CINAHL (via EBSCOhost), AMED (via OVID), EMBASE (via OVID), Global Health (via OVID), MEDLINE (via OVID), and PsycINFO (via OVID) databases. The search strategies for all databases are reproduced below.

MEDLINE

Database and platform: Medline (Ovid MEDLINE® Epub Ahead of Print, In-Process & Other Non-Indexed Citations, Ovid MEDLINE® Daily and Ovid MEDLINE®) 1946 to present

Search date: 28 May 2021

1. Arthritis, Psoriatic/
2. exp Arthritis/ and Psoriasis/
3. Enthesopathy/
4. psoriat$.ti,ab,kf.
5. enthesitis.ti,ab,kf.
6. enthesopath$.ti,ab,kf.
7. dactylitis.ti,ab,kf.
8. ((psorias$ or nail$) adj5 (arthrit$ or arthropath$ or monoarthr$ or oligoarthr$ or polyarthr$ or rheumat$)).ti,ab,kf.
9. ((psorias$ or nail$) adj5 (axial or peripheral or spondyl?arthr$ or arthropath$ or spondylitis or sacroili?ti$)).ti,ab,kf.
10. (psorias$ or nail$).ti,ab,kf. and (spondylarthropathies/ or Spondylarthritis/ or spondylitis, ankylosing/ or sacroiliitis/)
11. axPsA.ti,ab,kf.
12. pPsA.ti,ab,kf.
13. or/1-12
14. exp Smoking/
15. Smoking Cessation/
16. Smoking Reduction/
17. "Tobacco Use Cessation"/
18. Tobacco Smoking/
19. Vaping/
20. (smok$ or nicotin$ or tobacco$ or vape or vaping).ti,ab,kf.
21. or/14-20
22. Alcohol Drinking/
23. Alcoholism/
24. (alcohol$ or booz$ or liquor$).ti,ab,kf.
25. or/22-24
26. exp Substance-Related Disorders/
27. Illicit drugs/
28. "recreational drug$".ti,ab,kf.
29. ((substance$ or drug$ or solvent$ or narcotic$) adj2 (abus$ or depend$ or illegal$ or excess$ or use$ or addict$ or habit$ or usage or misuse)).ti,ab,kf.
30. or/26-29
31. exp Diet/
32. Diet, Healthy/
33. exp Diet Therapy/
34. (diet$ or nutriti$).ti,ab,kf.
35. or/31-34
36. Body Mass Index/
37. Obesity/
38. Overweight/
39. Weight loss/
40. Weight Reduction Programs/
41. Body Weight/
42. Body Weight Changes/
43. (obes$ or bariatri$ or slimm$).ti,ab,kf.
44. (weight$ adj2 (manage$ or health$ or loss$ or gain$ or change$ or over$ or reduct$ or control$ or modific$)).ti,ab,kf.
45. or/36-44
46. exp Exercise/
47. Physical Fitness/
48. Exercise Therapy/
49. (exercis$ or kinesiology).ti,ab,kf.
50. (physical$ adj1 (train$ or activit$ or move$ or work$ or exertion or "keep fit")).ti,ab,kf.
51. (train$ adj1 (endurance or resistance or autogenic)).ti,ab,kf.
52. (muscle$ adj1 (stretch$ or strength$ or "work-out$" or "work$ out")).ti,ab,kf.
53. or/46-52
54. exp Anxiety/
55. Mental Health/
56. exp Anxiety Disorders/
57. (anxi$ or "generali?ed anxiety disorder").ti,ab,kf.
58. or/54-57
59. exp Depression/
60. exp Mood Disorders/
61.(mood$ adj1 (low$ or reduc$)).ti,ab,kf.
62. (depress$ or miser$ or hopelessness or unhappy or sadness).ti,ab,kf.
63. or/59-62
64. Stress, Psychological/
65. (stress$ or pressure or tension$ or hassle or strain$ or distress or upset$).ti,ab,kf.
66. or/64-65
67. Pain Management/
68. (pain$ adj1 (control$ or relief$ or reduc$ or manage$ or alleviat$ or lesson$)).ti,ab,kf.
69. or/67-68
70. exp Sleep/
71. (sleep$ or rest$ or nap$ or slumber or drows$ or insomnia).ti,ab,kf.
72. or/70-71
73. Fatigue/
74. Sleepiness/
75. (fatigu$ or tired$ or weary or weariness or exhaust$ or lethargy or drain$ or "flare-up$" or "flare up" or flare$).ti,ab,kf.
76. or/73-75
77. Medication Adherence/
78. "Treatment Adherence and Compliance"/
79. (medic$ adj1 ("non-adheren$" or nonadheren$ or persisten$ or "non-complian$" or noncomplian$ or compliance or comply)).ti,ab,kf.
80. (drug$ adj1 ("non-adheren$" or nonadheren$ or persisten$ or "non-complian$" or noncomplian$ or compliance or comply)).ti,ab,kf.
81. or/77-80
82. exp Complementary Therapies/
83. ("complementary medicine" or "complementary therap$").ti,ab,kf.
84. (medicine adj1 (natural$ or folk or holist$ or unconvention$ or alternat$ or herb$ or faith or unorthodox or heal$)).ti,ab,kf.
85. ("traditional Chinese medicine" or "Chinese traditional medicine" or "Chinese herbal medicine").ti,ab,kf.
86. ("alternative therap$" or "faith heal$" or homeopath$ or chiropract$ or osteopath$ or "cranial sacral therapy").ti,ab,kf.
87. ((therap$ or remed$) adj1 (holist$ or herb$)).ti,ab,kf.
88. or/82-87
89. ((adjunct$ or adjuvant$) adj1 (therap$ or care or treatment$)).ti,ab,kf.
90. "add-on therapy".ti,ab,kf.
91. or/89-90
92. Acupuncture Therapy/
93. Physical Therapy Modalities/
94. Mind-Body Therapies/
95. Motivational Interviewing/
96. exp Cognitive Behavioral Therapy/
97. (acupunctur$ or yoga or hypnosis or qigong or meditate$ or meditation or "Tai ji" or "Tai Chi" or Reiki or "mental healing" or psychodrama$ or psychophysiology).ti,ab,kf.
98. ((psycholog$ or psycotherap$) adj1 (biofeedback or imagery)).ti,ab,kf.
99. (exercise$ adj1 breath$).ti,ab,kf.
100. (therap$ adj2 (touch or behavio$ or "acceptance and commitment" or cognitive or animal or dance or exercise or "extracorporeal shockwave" or CBT or "compassion focus$" or psycholog$)).ti,ab,kf.
101. "motivational interview$".ti,ab,kf.
102. (reflexotherapy or reflexology or mindfulness or "cognitive remediation" or "spinal cord stimulation" or "transcutaneous electric nerve stimulation" or TENS or "dry needling").ti,ab,kf.
103. (manipulat$ adj1 (spin$ or osteopath$ or musculoskelet$ or orthop$)).ti,ab,kf.
104. (("non pharmacologic$" or "non-pharmacologic$" or nonpharmacologic$) adj1 (treatment$ or therap$)).ti,ab,kf.
105. (therap$ adj1 (relax$ or massag$ or hydro$ or music$ or physical or physio$ or music$ or spirit$)).ti,ab,kf.
106. "psycholog$ intervention$".ti,ab,kf.
107. or/92-106
108. Sexual Behavior/
109. Sex Counseling/
110. Sexual Dysfunction, Physiological/
111. intima$.ti,ab,kf.
112. (relation$ adj1 (personal or close$ or caring or familiar$ or affection$)).ti,ab,kf.
113. (emotional$ adj1 (close$ or attachment or together$)).ti,ab,kf.
114. or/108-113
115. Social Isolation/
116. exp Psychology, Social/
117. (friend$ or "social relations$" or isolat$ or lonely or loneliness or work or job or employment or career or travel$ or holiday$ or vacation$ or journey$ or excursion$).ti,ab,kf.
118. (relation$ adj3 (friend$ or famil$ or wife$ or husband$ or spouse$ or partner$ or colleague$)).ti,ab,kf.
119. (friend$ adj1 (girl$ or boy$)).ti,ab,kf.
120. or/115-119
121. "Appointments and Schedules"/
122. exp Patient Acceptance of Health Care/
123. "Referral and Consultation"/
124. "Physician-Patient Relations"/
125. ((health$ or outpatient or clinic) adj1 (attendance or appointment$)).ti,ab,kf.
126. (accept$ adj3 diagnos$).ti,ab,kf.
127. "doctor patient communication".ti,ab,kf.
128. ((consultat$ or communicat$) adj2 (doctor$ or physician$ or medic or medics or nurse$ or physio$ or "healthcare profession$")).ti,ab,kf.
129. or/121-128
130. "Quality of Life"/
131. Psychosocial Functioning/
132. Life Style/
133. Healthy Lifestyle/
134. Health Behavior/
135. Personal satisfaction/
136. Patient satisfaction/
137. "Activities of Daily Living"/
138. "Quality-Adjusted Life Years"/
139. Social Support/
140. "quality of life".ti,ab,kf.
141. "life quality".ti,ab,kf.
142. ((patient or personal) adj1 (satisfaction or autonomy or preference$ or experience$)).ti,ab,kf.
143. "activities of daily living".ti,ab,kf.
144. "quality adjusted life year$".ti,ab,kf.
145. (health$ adj1 (life$ or behavio$)).ti,ab,kf.
146. (psycho$ adj1 function$).ti,ab,kf.
147. or/130-146
148. 21 or 25 or 30 or 35 or 45 or 53 or 58 or 63 or 66 or 69 or 72 or 76 or 81 or 88 or 91 or 107 or 114 or 120 or 129 or 147
149. 13 and 148
150. Limit 149 to yr="2010-2021"

EMBASE

Database and platform: EMBASE (via OVID) 1974 to present

Search date: 28 May 2021

1. Psoriatic Arthritis/
2. exp Arthritis/ and Psoriasis/
3. Enthesopathy/
4. psoriat$.ti,ab,kw.
5. enthesitis.ti,ab,kw.
6. enthesopath$.ti,ab,kw.
7. dactylitis.ti,ab,kw.
8. ((psorias$ or nail$) adj5 (arthrit$ or arthropath$ or monoarthr$ or oligoarthr$ or polyarthr$ or rheumat$)).ti,ab,kw.
9. ((psorias$ or nail$) adj5 (axial or peripheral or spondyl?arthr$ or arthropath$ or spondylitis or sacroili?ti$)).ti,ab,kw.
10. (psorias$ or nail$).ti,ab,kw. and (spondyloarthropathy/ or Spondylarthritis/ or spondylitis ankylosing/ or sacroiliitis/)
11. axPsA.ti,ab,kw.
12. pPsA.ti,ab,kw.
13. or/1-12
14. exp Smoking/
15. Smoking Cessation/
16. Smoking Reduction/
17. Vaping/
18. Electronic cigarette/
19. (smok$ or nicotin$ or tobacco$ or vape or vaping).ti,ab,kw.
20. or/14-19
21. Drinking Behavior/
22. Alcoholism/
23. (alcohol$ or booz$ or liquor$).ti,ab,kw.
24. or/21-23
25. Drug Dependence/
26. Substance Abuse/
27. Illicit Drug/
28. ((substance$ or drug$ or solvent$ or narcotic$) adj2 (abus$ or depend$ or illegal$ or excess$ or use$ or addict$ or habit$ or usage or misuse or illicit or recreation$)).ti,ab,kw.
29. or/25-28
30. Exp Diet/
31. exp Diet Therapy/
32. (diet$ or nutriti$).ti,ab,kw.
33. or/30-32
34. Body Mass/
35. Obesity/
36. Body Weight Loss/
37. Weight Loss Program/
38. Body Weight/
39. Body Weight Change/
40. (obes$ or bariatri$ or slimm$ or overweight).ti,ab,kw.
41. (weight$ adj2 (manage$ or health$ or loss$ or gain$ or change$ or over$ or reduct$ or control$ or modific$)).ti,ab,kw.
42. or/34-41
43. exp Exercise/
44. Fitness/
45. exp Kinesiotherapy/
46. (exercis$ or kinesiology).ti,ab,kw.
47. (physical$ adj1 (train$ or activit$ or move$ or work$ or exertion or "keep fit")).ti,ab,kw.
48. (train$ adj1 (endurance or resistance or autogenic)).ti,ab,kw.
49. (muscle$ adj1 (stretch$ or strength$ or "work-out$" or "work$ out")).ti,ab,kw.
50. or/43-49
51. exp Anxiety/
52. exp Mental Health/
53. exp Anxiety Disorder/
54. (anxi$ or "generali?ed anxiety disorder").ti,ab,kw.
55. or/51-54
56. exp Depression/
57. Mood Disorder/
58.(mood$ adj1 (low$ or reduc$)).ti,ab,kw.
59. (depress$ or miser$ or hopelessness or unhappy or sadness).ti,ab,kw.
60. or/56-59
61. Mental stress/
62. (stress$ or pressure or tension$ or hassle or strain$ or distress or upset$).ti,ab,kw.
63. or/61-62
64. (pain$ adj1 (control$ or relief$ or reduc$ or manage$ or alleviat$ or lesson$)).ti,ab,kw.
65. exp Sleep/
66. (sleep$ or rest$ or nap$ or slumber or drows$ or insomnia).ti,ab,kw.
67. or/65-66
68. Fatigue/
69. Exhaustion/
70. (fatigu$ or tired$ or weary or weariness or exhaust$ or lethargy or drain$ or "flare-up$" or "flare up" or flare$).ti,ab,kw.
71. or/68-70
72. Medication Compliance/
73. Patient Compliance/
74. (medic$ adj1 ("non-adheren$" or nonadheren$ or persisten$ or "non-complian$" or noncomplian$ or compliance or comply)).ti,ab,kw.
75. (drug$ adj1 ("non-adheren$" or nonadheren$ or persisten$ or "non-complian$" or noncomplian$ or compliance or comply)).ti,ab,kw.
76. or/72-75
77. exp Alternative Medicine/
78. Chinese Medicine/
79. Traditional Medicine/
80. ("complementary medicine" or "complementary therap$").ti,ab,kw.
81. (medicine adj1 (natural$ or folk or holist$ or unconvention$ or alternat$ or herb$ or faith or unorthodox or heal$)).ti,ab,kw.
82. ("traditional Chinese medicine" or "Chinese traditional medicine" or "Chinese herbal medicine").ti,ab,kw.
83. ("alternative therap$" or "faith heal$" or homeopath$ or chiropract$ or osteopath$ or "cranial sacral therapy").ti,ab,kw.
84. ((therap$ or remed$) adj1 (holist$ or herb$)).ti,ab,kw.
85. or/77-84
86. ((adjunct$ or adjuvant$) adj1 (therap$ or care or treatment$)).ti,ab,kw.
87. "add-on therapy".ti,ab,kw.
88. or/86-87
89. Acupuncture/
90. Physiotherapy/
91. Psychotherapy/
92. Motivational interviewing/
93. Cognitive Behavioral Therapy/
94. (acupunctur$ or yoga or hypnosis or qigong or meditate$ or meditation or "Tai ji" or "Tai Chi" or Reiki or "mental healing" or psychodrama$ or psychophysiology).ti,ab,kw.
95. ((psycholog$ or psycotherap$) adj1 (biofeedback or imagery)).ti,ab,kw.
96. (exercise$ adj1 breath$).ti,ab,kw.
97. "motivational interview$".ti,ab,kw.
98. (therap$ adj2 (touch or behavio$ or "acceptance and commitment" or cognitive or animal or dance or exercise or "extracorporeal shockwave" or CBT or "compassion focus$" or psycholog$)).ti,ab,kw.
99. (reflexotherapy or reflexology or mindfulness or "cognitive remediation" or "spinal cord stimulation" or "transcutaneous electric nerve stimulation" or TENS or "dry needling").ti,ab,kw.
100. (manipulat$ adj1 (spin$ or osteopath$ or musculoskelet$ or orthop$)).ti,ab,kw.
101. (("non pharmacologic$" or "non-pharmacologic$" or nonpharmacologic$) adj1 (treatment$ or therap$)).ti,ab,kw.
102. (therap$ adj1 (relax$ or massag$ or hydro$ or music$ or physical or physio$ or music$ or spirit$)).ti,ab,kw.
103. "psycholog$ intervention$".ti,ab,kw.
104. or/89-103
105. Sexual Behavior/
106. Sexual Counseling/
107. Sexual Dysfunction/
108. intima$.ti,ab,kw.
109. (relation$ adj1 (personal or close$ or caring or familiar$ or affection$)).ti,ab,kw.
110. (emotional$ adj1 (close$ or attachment or together$)).ti,ab,kw.
111. or/105-110
112. exp Social Isolation/
113. Social interaction/
114. Social Psychology/
115. (friend$ or "social relations$" or isolat$ or lonely or loneliness or work or job or employment or career or travel$ or holiday$ or vacation$ or journey$ or excursion$).ti,ab,kw.
116. (relation$ adj3 (friend$ or famil$ or wife$ or husband$ or spouse$ or partner$ or colleague$)).ti,ab,kw.
117. (friend$ adj1 (girl$ or boy$)).ti,ab,kw.
118. or/112-117
119. Doctor Patient Relation/
120. Patient Referral/
121. ((health$ or outpatient or clinic) adj1 (attendance or appointment$)).ti,ab,kw.
122. (accept$ adj3 diagnos$).ti,ab,kw.
123. "doctor patient communication".ti,ab,kw.
124. ((consultat$ or communicat$) adj2 (doctor$ or physician$ or medic or medics or nurse$ or physio$ or "healthcare profession$")).ti,ab,kw.
125. or/119-124
126. exp "quality of life"/
127. social psychology/
128. exp lifestyle/
129. exp lifestyle modification/
130. "lifestyle and related phenomena"/
131. exp health behavior/
132. patient satisfaction/
133. satisfaction/
134. daily life activity/
135. social support/
136. "quality of life".ti,ab,kw.
137. "life quality".ti,ab,kw.
138. ((patient or personal) adj1 (satisfaction or autonomy or preference$ or experience$)).ti,ab,kw.
139. "activities of daily living".ti,ab,kw.
140. "quality adjusted life year$".ti,ab,kw.
141. (health$ adj1 (life$ or behavio$)).ti,ab,kw.
142. (psycho$ adj1 (function$)).ti,ab,kw.
143. or/126-142
144. 20 or 24 or 29 or 33 or 42 or 50 or 55 or 60 or 63 or 64 or 67 or 71 or 76 or 85 or 88 or 104 or 111 or 118 or 125 or 143
145. 13 and 144
146. Limit 145 to yr="2010-2021"

CINAHL

Database and platform: CINAHL (Cumulative Index to Nursing and Allied Health Literature ) (via EBSCOhost)

Search date: 28 May 2021

1. (MH "Arthritis, Psoriatic")
2. ((MH "Arthritis+") AND (MH "Psoriasis"))
3. (MH "Enthesopathy")
4. (TI psoriat* OR AB psoriat*)
5. (TI enthesitis OR AB enthesitis)
6. (TI enthesopath* OR AB enthesopath*)
7. (TI dactylitis OR AB dactylitis)
8. (TI (psorias* or nail*) N5 (arthrit* or arthropath* or monoarthr* or oligoarthr* or polyarthr* or rheumat*)) OR (AB (psorias* or nail*) N5 (arthrit* or arthropath* or monoarthr* or oligoarthr* or polyarthr* or rheumat*))
9. (TI (psorias* or nail*) N5 (axial or peripheral or spondyl?arthr* or arthropath* or spondylitis or sacroili?ti*)) OR (AB (psorias* or nail*) N5 (axial or peripheral or spondyl?arthr* or arthropath* or spondylitis or sacroili?ti*))
10. (TI (psorias* or nail*) AND (MH "Spondylarthropathies" OR MH "spondylarthritis" OR MH "spondylitis, ankylosing")) OR (AB (psorias* or nail*) AND (MH "Spondylarthropathies" OR MH "spondylarthritis" OR MH "spondylitis, ankylosing"))
11. (TI axPsA OR AB axPsA)
12. (TI pPsA OR AB pPsA)
13. S1 OR S2 OR S3 OR S4 OR S5 OR S6 OR S7 OR S8 OR S9 OR S10 OR S11 OR S12
14. (MH "Smoking+")
15. (MH "Vaping")
16. (TI smok* or nicotin* or tobacco* or vape or vaping) OR (AB smok* or nicotin* or tobacco* or vape or vaping)
17. S14 OR S15 OR S16
18. (MH "Alcohol Drinking+")
19. (MH "Alcoholism")
20. (TI alcohol* or booz* or liquor*) OR (AB alcohol* or booz* or liquor*)
21. S18 OR S19 OR S20
22. (MH "Substance Use Disorders+")
23. (MH "Street Drugs+")
24. (TI "recreational drug*") OR (AB "recreational drug*")
25. (TI (substance* or drug* or solvent* or narcotic*) N2 (abus* or depend* or illegal* or excess* or use* or addict* or habit* or usage or misuse)) OR (AB (substance* or drug* or solvent* or narcotic*) N2 (abus* or depend* or illegal* or excess* or use* or addict* or habit* or usage or misuse))
26. S22 OR S23 OR S24 OR S25
27. (MH "Diet+")
28. (MH "Diet Therapy+")
29. (TI diet* or nutriti*) OR (AB diet* or nutriti*)
30. S27 OR S28 OR S29
31. (MH "Body Mass Index")
32. (MH "Obesity")
33. (MH "Weight loss")
34. (MH "Weight Reduction Programs")
35. (MH "Body Weight")
36. (MH "Body Weight Changes")
37. (TI obes* or bariatri* or slimm*) OR (AB obes* or bariatri* or slimm*)
38. (TI weight* N2 (manage* or health* or loss* or gain* or change* or over* or reduct* or control* or modific*)) OR (AB weight* N2 (manage* or health* or loss* or gain* or change* or over* or reduct* or control* or modific*))
39. S31 OR S32 OR S33 OR S34 OR S35 OR S36 OR S37 OR S38
40. (MH "Exercise+")
41. (MH "Physical Fitness")
42. (MH "Therapeutic Exercise+")
43. (TI exercis* or kinesiology) OR (AB exercis* or kinesiology)
44. (TI physical* N1 (train* or activit* or move* or work* or exertion or "keep fit")) OR (AB physical* N1 (train* or activit* or move* or work* or exertion or "keep fit"))
45. (TI train* N1 (endurance or resistance or autogenic)) OR (AB train* N1 (endurance or resistance or autogenic))
46. (TI muscle* N1 (stretch* or strength* or "work-out*" or "work* out")) OR (AB muscle* N1 (stretch* or strength* or "work-out*" or "work* out"))
47. S40 OR S41 OR S42 OR S43 OR S44 OR S45 OR S46
48. (MH "Anxiety+")
49. (MH "Mental Health")
50. (MH "Anxiety Disorders+")
51. (TI anxi* or "generali?ed anxiety disorder") OR (AB anxi* or "generali?ed anxiety disorder")
52. S48 OR S49 OR S50 OR S51
53. (MH "Depression")
54. (MH "Affective Disorders")
55. (TI mood* N1 (low* or reduc*)) OR (AB mood* N1 (low* or reduc*))
56. (TI depress* or miser* or hopelessness or unhappy or sadness) OR (AB depress* or miser* or hopelessness or unhappy or sadness)
57. S53 OR S54 OR S55 OR S56
58. (MH "Stress, Psychological")
59. (TI stress* or pressure or tension* or hassle or strain* or distress or upset*) OR (AB stress* or pressure or tension* or hassle or strain* or distress or upset*)
60. S58 OR S59
61. (MH "Pain Management")
62. (TI pain* N1 (control* or relief* or reduc* or manage* or alleviat* or lesson*)) OR (AB pain* N1 (control* or relief* or reduc* or manage* or alleviat* or lesson*))
63. S61 OR S62
64. (MH "Sleep+")
65. (TI sleep* or rest* or nap* or slumber or drows* or insomnia) OR (AB sleep* or rest* or nap* or slumber or drows* or insomnia)
66. S64 OR S65
67. (MH "Fatigue")
68. (MH "Sleepiness")
69. (TI fatigu* or tired* or weary or weariness or exhaust* or lethargy or drain* or "flare-up*" or "flare up*" or flare*) OR (AB fatigu* or tired* or weary or weariness or exhaust* or lethargy or drain* or "flare-up*" or "flare up*" or flare*)
70. S67 OR S68 OR S69
71. (MH "Medication Compliance")
72. (TI medic* N1 ("non-adheren*" or nonadheren* or persisten* or "non-complian*" or noncomplian* or compliance or comply)) OR (AB medic* N1 ("non-adheren*" or nonadheren* or persisten* or "non-complian*" or noncomplian* or compliance or comply))
73. (TI drug* N1 ("non-adheren*" or nonadheren* or persisten* or "non-complian*" or noncomplian* or compliance or comply)) OR (AB drug* N1 ("non-adheren*" or nonadheren* or persisten* or "non-complian*" or noncomplian* or compliance or comply))
74. S71 OR S72 OR S73
75. (MH "Alternative Therapies+")
76. (TI "complementary medicine" or "complementary therap*") OR (AB "complementary medicine" or "complementary therap*")
77. (TI medicine N1 (natural* or folk or holist* or unconvention* or alternat* or herb* or faith or unorthodox or heal*)) OR (AB medicine N1 (natural* or folk or holist* or unconvention* or alternat* or herb* or faith or unorthodox or heal*))
78. (TI "traditional Chinese medicine" or "Chinese traditional medicine" or "Chinese herbal medicine") OR (AB "traditional Chinese medicine" or "Chinese traditional medicine" or "Chinese herbal medicine")
79. (TI "alternative therap*" or "faith heal*" or homeopath* or chiropract* or osteopath* or "cranial sacral therapy") OR (AB "alternative therap*" or "faith heal*" or homeopath* or chiropract* or osteopath* or "cranial sacral therapy")
80. (TI (therap* or remed*) N1 (holist* or herb*)) OR (AB (therap* or remed*) N1 (holist* or herb*))
81. S75 OR S76 OR S77 OR S78 OR S79 OR S80
82. (TI (adjunct* or adjuvant*) N1 (therap* or care or treatment*)) OR (AB (adjunct* or adjuvant*) N1 (therap* or care or treatment*))
83. (TI "add-on therapy") OR (AB "add-on therapy")
84. S82 OR S83
85. (MH "Acupuncture+")
86. (MH "Physical Therapy+")
87. (MH "Mind Body Techniques+")
88. (MH "Motivational Interviewing")
89. (MH "Cognitive Therapy")
90. (TI acupunctur* or yoga or hypnosis or qigong or meditate* or meditation or "Tai ji" or "Tai Chi" or Reiki or "mental healing" or psychodrama* or psychophysiology) OR (AB acupunctur* or yoga or hypnosis or qigong or meditate* or meditation or "Tai ji" or "Tai Chi" or Reiki or "mental healing" or psychodrama* or psychophysiology)
91. (TI (psycholog* or psycotherap*) N1 (biofeedback or imagery)) OR (AB (psycholog* or psycotherap*) N1 (biofeedback or imagery))
92. (TI exercise* N1 breath*) OR (AB exercise* N1 breath*)
93. (TI therap* N2 (touch or behavio* or "acceptance and commitment" or cognitive or animal or dance or exercise or "extracorporeal shockwave" or CBT or "compassion focus$" or psycholog$)) OR (AB therap* N2 (touch or behavio* or "acceptance and commitment" or cognitive or animal or dance or exercise or "extracorporeal shockwave" or CBT))
94. (TI "motivational interview*") OR (AB "motivational interview*")
95. (TI reflexotherapy or reflexology or mindfulness or "cognitive remediation" or "spinal cord stimulation" or "transcutaneous electric nerve stimulation" or TENS or "dry needling") OR (AB reflexotherapy or reflexology or mindfulness or "cognitive remediation" or "spinal cord stimulation" or "transcutaneous electric nerve stimulation" or TENS or "dry needling")
96. (TI manipulat* N1 (spin* or osteopath* or musculoskelet* or orthop*)) OR (AB manipulat* N1 (spin* or osteopath* or musculoskelet* or orthop*))
97. (TI ("non pharmacologic*" or "non-pharmacologic*" or nonpharmacologic*) N1 (treatment* or therap*)) OR (AB ("non pharmacologic*" or "non-pharmacologic*" or nonpharmacologic*) N1 (treatment* or therap*))
98. (TI therap* N1 (relax* or massag* or hydro* or music* or physical or physio* or music* or spirit*)) OR (AB therap* N1 (relax* or massag* or hydro* or music* or physical or physio* or music* or spirit*))
99. (TI "psycholog* intervention*") OR (AB "psycholog* intervention*")
100. S85 OR S86 OR S87 OR S88 OR S89 OR S90 OR S91 OR S92 OR S93 OR S94 OR S95 OR S96 OR S97 OR S98 OR S99
101. (MH "Sexual Counseling")
102. (MH "Sexual Dysfunction, Male")
103. (MH "Sexual Dysfunction, Female")
104. (TI intima* OR AB intima*)
105. (TI relation* N1 (personal or close* or caring or familiar* or affection*)) OR (AB relation* N1 (personal or close* or caring or familiar* or affection*))
106. (TI emotional* N1 (close* or attachment or together*)) OR (AB emotional* N1 (close* or attachment or together*))
107. S101 OR S102 OR S103 OR S104 OR S105 OR S106
108. (MH "Social Isolation+")
109. (MH "Psychology, Social+")
110. (TI friend* or "social relations*" or isolat* or lonely or loneliness or work or job or employment or career or travel* or holiday* or vacation* or journey* or excursion*) OR (AB friend* or "social relations*" or isolat* or lonely or loneliness or work or job or employment or career or travel* or holiday* or vacation* or journey* or excursion*)
111. (TI relation* N3 (friend* or famil* or wife* or husband* or spouse* or partner* or colleague*)) OR (AB relation* N3 (friend* or famil* or wife* or husband* or spouse* or partner* or colleague*))
112. (TI friend* N1 (girl* or boy*)) OR (AB friend* N1 (girl* or boy*))
113. S108 OR S109 OR S110 OR S111 OR S112
114. (MH "Appointments and Schedules")
115. (MH "Referral and Consultation")
116. (TI (health* or outpatient or clinic) N1 (attendance or appointment*)) OR (AB (health* or outpatient or clinic) N1 (attendance or appointment*))
117. (TI accept* N3 diagnos*) OR (AB accept* N3 diagnos*)
118. (TI "doctor patient communication") OR (AB "doctor patient communication")
119. (TI (consultat* or communicat*) N2 (doctor* or physician* or medic or medics or nurs* or physio* or "healthcare profession*")) OR (AB (consultat* or communicat*) N2 (doctor* or physician* or medic or medics or nurs* or physio* or "healthcare profession*"))
120. S114 OR S115 OR S116 OR S117 OR S118 OR S119
121. (MH "Quality of Life+")
122. (MH "Life Style+")
123. (MH "Personal Satisfaction")
124. (MH "Patient Satisfaction")
125. (MH "Activities of Daily Living")
126. (MH "Quality-Adjusted Life Years")
127. (MH "Support, Psychosocial")
128. (TI "quality of life") OR (AB "quality of life")
129. (TI "life quality") OR (AB "life quality")
130. (TI (patient or personal) N1 (satisfaction or autonomy or preference* or experience*)) OR (AB (patient or personal) N1 (satisfaction or autonomy or preference* or experience*))
131. (TI "activities of daily living") OR (AB "activities of daily living")
132. (TI “quality adjusted life year*”) OR (AB “quality adjusted life year*”)
133. (TI (health* N1 (life* or behavio*)) OR (AB (health* N1 (life* or behavio*))
134. (TI psycho* N1 function*) OR (AB psycho* N1 function*)
135. S121 OR S122 OR S123 OR S124 OR S125 OR S126 OR S127 OR S128 OR S129 OR S130 OR S131 OR S132 OR S133 OR S134
136. S17 OR S21 OR S26 OR S30 OR S39 OR S47 OR S52 OR S57 OR S60 OR S63 OR S66 OR S70 OR S74 OR S81 OR S84 OR S100 OR S107 OR S113 OR S120 OR S135
137. S13 and S136
138. PY "2010-2021"
139. S137 AND S138

AMED

Database and platform: AMED (Allied and Complementary Medicine) 1985 to present (via OVID)

Search date: 28 May 2021

1. exp Arthritis/ and Psoriasis/
2. enthesopathy.ti,ab.
3. psoriat$.ti,ab.
4. enthesitis.ti,ab.
5. enthesopath$.ti,ab.
6. dactylitis.ti,ab.
7. ((psorias$ or nail$) adj5 (arthrit$ or arthropath$ or monoarthr$ or oligoarthr$ or polyarthr$ or rheumat$)).ti,ab.
8. ((psorias$ or nail$) adj5 (axial or peripheral or spondyl?arthr$ or arthropath$ or spondylitis or sacroili?ti$)).ti,ab.
9. ((psorias$ or nail$).ti,ab. and Spondylitis Ankylosing/)
10. axPsA.ti,ab.
11. pPsA.ti,ab.
12. or/1-11
13. exp Smoking/
14. (smok$ or nicotin$ or tobacco$ or vape or vaping).ti,ab.
15. or/13-14
16. Alcohol Drinking/
17. Alcoholism/
18. (alcohol$ or booz$ or liquor$).ti,ab.
19. or/16-18
20. exp Substance Related Disorders/
21. ((substance$ or drug$ or solvent$ or narcotic$) adj2 (abus$ or depend$ or illegal$ or excess$ or use$ or addict$ or habit$ or usage or misuse or illicit or recreation$)).ti,ab.
22. or/20-21
23. exp Diet/
24. exp Diet Therapy/
25. Nutrition therapy/
26. (diet$ or nutriti$).ti,ab.
27. or/23-26
28. Body Mass Index/
29. Obesity/
30. Weight loss/
31. Body Weight/
32. overweight.ti,ab.
33. (obes$ or bariatri$ or slimm$).ti,ab.
34. (weight$ adj2 (manage$ or health$ or loss$ or gain$ or change$ or over$ or reduct$ or control$ or modific$)).ti,ab.
35. or/28-34
36. exp Exercise/
37. Physical Fitness/
38. Exercise Therapy/
39. (exercis$ or kinesiology).ti,ab.
40. (physical$ adj1 (train$ or activit$ or move$ or work$ or exertion or "keep fit")).ti,ab.
41. (train$ adj1 (endurance or resistance or autogenic)).ti,ab.
42. (muscle$ adj1 (stretch$ or strength$ or "work-out$" or "work$ out")).ti,ab.
43. or/36-42
44. Anxiety/
45. Mental Health/
46. exp Anxiety Disorders/
47. (anxi$ or "generali?ed anxiety disorder").ti,ab.
48. or/44-47
49. Depression/
50. "mood disorder$".ti,ab.
51.(mood$ adj1 (low$ or reduc$)).ti,ab.
52. (depress$ or miser$ or hopelessness or unhappy or sadness).ti,ab.
53. or/49-52
54. Stress Psychological/
55. (stress$ or pressure or tension$ or hassle or strain$ or distress or upset$).ti,ab.
56. or/54-55
57. "pain management".ti,ab.
58. (pain$ adj1 (control$ or relief$ or reduc$ or manage$ or alleviat$ or lesson$)).ti,ab.
59. or/57-58
60. exp Sleep/
61. (sleep$ or rest$ or nap$ or slumber or drows$ or insomnia).ti,ab.
62. or/60-61
63. exp Fatigue/
64. (fatigu$ or tired$ or weary or weariness or exhaust$ or lethargy or drain$ or "flare-up$" or "flare up" or flare$ or sleepiness).ti,ab.
65. or/63-64
66. Patient compliance/
67. ("medication adherence" or "medication compliance").ti,ab.
68. ("treatment adherence" or "treatment compliance").ti,ab.
69. (medic$ adj1 ("non-adheren$" or nonadheren$ or persisten$ or "non-complian$" or noncomplian$ or compliance or comply)).ti,ab.
70. (drug$ adj1 ("non-adheren$" or nonadheren$ or persisten$ or "non-complian$" or noncomplian$ or compliance or comply)).ti,ab.
71. or/66-70
72. exp Complementary Therapies/
73. ("complementary medicine" or "complementary therap$").ti,ab.
74. (medicine adj1 (natural$ or folk or holist$ or unconvention$ or alternat$ or herb$ or faith or unorthodox or heal$)).ti,ab.
75. ("traditional Chinese medicine" or "Chinese traditional medicine" or "Chinese herbal medicine").ti,ab.
76. ("alternative therap$" or "faith heal$" or homeopath$ or chiropract$ or osteopath$ or "cranial sacral therapy").ti,ab.
77. ((therap$ or remed$) adj1 (holist$ or herb$)).ti,ab.
78. or/72-77
79. ((adjunct$ or adjuvant$) adj1 (therap$ or care or treatment$)).ti,ab.
80. "add-on therapy".ti,ab.
81. or/79-80
82. Physical Therapy Modalities/
83. (acupunctur$ or yoga or hypnosis or qigong or meditate$ or meditation or "Tai ji" or "Tai Chi" or Reiki or "mental healing" or psychodrama$ or psychophysiology).ti,ab.
84. ((psycholog$ or psycotherap$) adj1 (biofeedback or imagery)).ti,ab.
85. (exercise$ adj1 breath$).ti,ab.
86. (therap$ adj2 (touch or behavio$ or "acceptance and commitment" or cognitive or animal or dance or exercise or "extracorporeal shockwave" or CBT or "compassion focus$" or psycholog$)).ti,ab.
87. "motivational interview$".ti,ab.
88. (reflexotherapy or reflexology or mindfulness or "cognitive remediation" or "spinal cord stimulation" or "transcutaneous electric nerve stimulation" or TENS or "dry needling").ti,ab.
89. (therap$ adj2 (touch or behavio$ or "acceptance and commitment" or cognitive or animal or dance or exercise or "extracorporeal shockwave" or CBT or "compassion focus$" or psycholog$)).ti,ab.
90. (reflexotherapy or reflexology or mindfulness or "cognitive remediation" or "spinal cord stimulation" or "transcutaneous electric nerve stimulation" or TENS or "dry needling").ti,ab.
91. (manipulat$ adj1 (spin$ or osteopath$ or musculoskelet$ or orthop$)).ti,ab.
92. (("non pharmacologic$" or "non-pharmacologic$" or nonpharmacologic$) adj1 (treatment$ or therap$)).ti,ab.
93. (therap$ adj1 (relax$ or massag$ or hydro$ or music$ or physical or physio$ or music$ or spirit$)).ti,ab.
94. "psycholog$ intervention$".ti,ab.
95. or/82-94
96. Sex Behavior/
97. Sex Counseling/
98. Sexual Dysfunctions/
99. intima$.ti,ab.
100. (relation$ adj1 (personal or close$ or caring or familiar$ or affection$)).ti,ab.
101. (emotional$ adj1 (close$ or attachment or together$)).ti,ab.
102. or/96-101
103. Social Isolation/
104. exp Psychology Social/
105. Employment/
106. (friend$ or "social relations$" or isolat$ or lonely or loneliness or work or job or employment or career or travel$ or holiday$ or vacation$ or journey$ or excursion$).ti,ab.
107. (relation$ adj3 (friend$ or famil$ or wife$ or husband$ or spouse$ or partner$ or colleague$)).ti,ab.
108. (friend$ adj1 (girl$ or boy$)).ti,ab.
109. or/103-108
110. "Referral and Consultation"/
111. exp Patient Acceptance of Health Care/
112. ((health$ or outpatient or clinic) adj1 (attendance or appointment$)).ti,ab.
113. (accept$ adj3 diagnos$).ti,ab.
114. "doctor patient communication".ti,ab.
115. ((consultat$ or communicat$) adj2 (doctor$ or physician$ or medic or medics or nurse$ or physio$ or "healthcare profession$")).ti,ab.
116. or/110-115
117. "Quality of life"/
118. exp Life style/
119. exp Health Behavior/
120. Patient satisfaction/
121. "Activities of Daily Living"/
122. Social Support/
123. "quality of life".ti,ab.
124. "life quality".ti,ab.
125. ((patient or personal) adj1 (satisfaction or autonomy or preference$ or experience$)).ti,ab.
126. "activities of daily living".ti,ab.
127. "quality adjusted life year$".ti,ab.
128. (health$ adj1 (life$ or behavio$)).ti,ab.
129. (psycho$ adj1 function$).ti,ab.
130. or/117-129
131. 15 or 19 or 22 or 27 or 35 or 43 or 48 or 53 or 56 or 59 or 62 or 65 or 71 or 78 or 81 or 95 or 102 or 109 or 116 or 130
132. 12 and 131
133. Limit 132 to yr="2010-2021"

Cochrane Central Register of Controlled Trials (CENTRAL)

Database and platform: Cochrane Library Central Register of Controlled Trials (CENTRAL) (via https://www.cochranelibrary.com/)

Search date: 28 May 2021

1. MeSH descriptor: [Arthritis, Psoriatic] explode all trees
2. MeSH descriptor: [Arthritis] explode all trees
3. MeSH descriptor: [Psoriasis] this term only
4. #2 and #3 in Trials
5. MeSH descriptor: [Enthesopathy] explode all trees
6. (psoriatic or enthesitis or enthesopathy or dactylitis):ti,ab,kw in Trials
7. ((psoriasis or nail) next/5 (arthritis or arthropathy or monoarthropathy or oligoarthropathy or polyarthritis or rheumatoid)):ti,ab,kw in Trials
8. ((psoriasis or nail) next/5 (axial or peripheral or spondylarthritis or arthropathy or sacroiliitis)):ti,ab,kw in Trials
9. (psorias$ or nail):ti,ab,kw in Trials
10. MeSH descriptor: [Spondylarthropathies] explode all trees
11. MeSH descriptor: [Spondylarthritis] explode all trees
12. MeSH descriptor: [Spondylitis] explode all trees
13. MeSH descriptor: [Ankylosis] explode all trees
14. MeSH descriptor: [Sacroiliitis] explode all trees
15. #10 or #11 or #12 or #13 or #14 in Trials
16. #9 and #15 in Trials
17. (axPsA or pPsA):ti,ab,kw in Trials
18. #1 or #4 or #5 or #6 or #7 or #8 or #16 or #17 with Publication Year from 2010 to 2021, in Trials

Global Health

Database and platform: Global Health (via OVID) 1973 to present

Search date: 28 May 2021

1. exp Arthritis/ and Psoriasis/
2. Enthesopathy/
3. psoriat$.ti,ab.
4. enthesitis.ti,ab.
5. enthesopath$.ti,ab.
6. dactylitis.ti,ab.
7. ((psorias$ or nail$) adj5 (arthrit$ or arthropath$ or monoarthr$ or oligoarthr$ or polyarthr$ or rheumat$)).ti,ab.
8. ((psorias$ or nail$) adj5 (axial or peripheral or spondyl?arthr$ or arthropath$ or spondylitis or sacroili?ti$)).ti,ab.
9. (psorias$ or nail$).ti,ab. and Ankylosing Spondylitis/
10. axPsA.ti,ab.
11. pPsA.ti,ab.
12. or/1-11
13. exp Tobacco Smoking/
14. Smoking Cessation/
15. Vaping/
16. (smok$ or nicotin$ or tobacco$ or vape or vaping).ti,ab.
17. or/13-16
18. Alcohol Intake/
19. Alcoholism/
20. (alcohol$ or booz$ or liquor$).ti,ab.
21. or/18-20
22. exp Substance Abuse/
23. ((substance$ or drug$ or solvent$ or narcotic$) adj2 (abus$ or depend$ or illegal$ or excess$ or use$ or addict$ or habit$ or usage or misuse or illicit or recreation$)).ti,ab.
24. or/22-23
25. exp Diets/
26. Diet/
27. Diet Treatment/
28. Dieting/
29. Nutrition/
30. (diet$ or nutriti$).ti,ab.
31. or/25-30
32. Body Mass Index/
33. Obesity/
34. Overweight/
35. Weight Losses/
36. Weight Loss Diets/
37. Weight Reduction/
38. Weight Control/
39. Body Weight/
40. Weight Gain/
41. (obes$ or bariatri$ or slimm$).ti,ab.
42. (weight$ adj2 (manage$ or health$ or loss$ or gain$ or change$ or over$ or reduct$ or control$ or modific$)).ti,ab.
43. or/32-42
44. Exercise/
45. Physical Activity/
46. Physical Fitness/
47. (exercis$ or kinesiology).ti,ab.
48. (physical$ adj1 (train$ or activit$ or move$ or work$ or exertion or "keep fit")).ti,ab.
49. (train$ adj1 (endurance or resistance or autogenic)).ti,ab.
50. (muscle$ adj1 (stretch$ or strength$ or "work-out$" or "work$ out")).ti,ab.
51. or/44-50
52. Anxiety/
53. Mental Health/
54. (anxi$ or "generali?ed anxiety disorder").ti,ab.
55. or/52-54
56. Depression/
57. (mood$ adj1 (low$ or reduc$ or disorder$)).ti,ab.
58. (depress$ or miser$ or hopelessness or unhappy or sadness).ti,ab.
59. or/56-58
60. Mental stress/
61. (stress$ or pressure or tension$ or hassle or strain$ or distress or upset$).ti,ab.
62. or/60-61
63. (pain$ adj1 (control$ or relief$ or reduc$ or manage$ or alleviat$ or lesson$)).ti,ab.
64. Sleep/
65. (sleep$ or rest$ or nap$ or slumber or drows$ or insomnia).ti,ab.
66. or/64-65
67. Fatigue/
68. Exhaustion/
69. (fatigu$ or tired$ or weary or weariness or exhaust$ or lethargy or drain$ or "flare-up$" or "flare up" or flare$).ti,ab.
70. or/67-69
71. Patient Compliance/
72. ("treatment adherence" or "treatment compliance" or "medication adherence" or "medication compliance").ti,ab.
73. (medic$ adj1 ("non-adheren$" or nonadheren$ or persisten$ or "non-complian$" or noncomplian$ or compliance or comply)).ti,ab.
74. (drug$ adj1 ("non-adheren$" or nonadheren$ or persisten$ or "non-complian$" or noncomplian$ or compliance or comply)).ti,ab.
75. or/71-74
76. exp Alternative Medicine/
77. exp Traditional Medicines/
78. Herbal Drugs/
79. exp Traditional Medicine/
80. Complementary Medicine/
81. ("complementary medicine" or "complementary therap$").ti,ab.
82. (medicine adj1 (natural$ or folk or holist$ or unconvention$ or alternat$ or herb$ or faith or unorthodox or heal$)).ti,ab.
83. ("traditional Chinese medicine" or "Chinese traditional medicine" or "Chinese herbal medicine").ti,ab.
84. ("alternative therap$" or "faith heal$" or homeopath$ or chiropract$ or osteopath$ or "cranial sacral therapy").ti,ab.
85. ((therap$ or remed$) adj1 (holist$ or herb$)).ti,ab.
86. or/76-85
87. Adjuvants/
88. ((adjunct$ or adjuvant$) adj1 (therap$ or care or treatment$)).ti,ab.
89. "add-on therapy".ti,ab.
90. or/87-89
91. Physical Therapy/
92. Psychotherapy/
93. (acupunctur$ or yoga or hypnosis or qigong or meditate$ or meditation or "Tai ji" or "Tai Chi" or Reiki or "mental healing" or psychodrama$ or psychophysiology).ti,ab.
94. ((psycholog$ or psycotherap$) adj1 (biofeedback or imagery)).ti,ab.
95. (exercise$ adj1 breath$).ti,ab.
96. (therap$ adj2 (touch or behavio$ or "acceptance and commitment" or cognitive or animal or dance or exercise or "extracorporeal shockwave" or CBT or "compassion focus$" or psycholog$)).ti,ab.
97. (reflexotherapy or reflexology or mindfulness or "cognitive remediation" or "spinal cord stimulation" or "transcutaneous electric nerve stimulation" or TENS or "dry needling").ti,ab.
98. (manipulat$ adj1 (spin$ or osteopath$ or musculoskelet$ or orthop$)).ti,ab.
99. (("non pharmacologic$" or "non-pharmacologic$" or nonpharmacologic$) adj1 (treatment$ or therap$)).ti,ab.
100. (therap$ adj1 (relax$ or massag$ or hydro$ or music$ or physical or physio$ or music$ or spirit$)).ti,ab.
101. "motivational interview$".ti,ab.
102. "psycholog$ intervention$".ti,ab.
103. or/91-102
104. Sexual Behaviour/
105. (sex$ adj1 dysfunction$).ti,ab.
106. intima$.ti,ab.
107. (relation$ adj1 (personal or close$ or caring or familiar$ or affection$)).ti,ab.
108. (emotional$ adj1 (close$ or attachment or together$)).ti,ab.
109. or/104-108
110. Social Isolation/
111. Social Interaction/
112. Loneliness/
113. Interpersonal Relations/
114. Holidays/
115. Employment/
116. Occupations/
117. Livelihoods/
118. (social adj1 psycholog$).ti,ab.
119. (friend$ or "social relations$" or isolat$ or lonely or loneliness or work or job or employment or career or travel$ or holiday$ or vacation$ or journey$ or excursion$).ti,ab.
120. (relation$ adj3 (friend$ or famil$ or wife$ or husband$ or spouse$ or partner$ or colleague$)).ti,ab.
121. (friend$ adj1 (girl$ or boy$)).ti,ab.
122. or/110-121
123. exp Patients/
124. ((health$ or outpatient or clinic) adj1 (attendance or appointment$)).ti,ab.
125. (accept$ adj3 diagnos$).ti,ab.
126. "doctor patient communication".ti,ab.
127. ((consultat$ or communicat$) adj2 (doctor$ or physician$ or medic or medics or nurse$ or physio$ or "healthcare profession$")).ti,ab.
128. or/123-127
129. exp "quality of life"/
130. lifestyle/
131. health behaviour/
132. "quality of life".ti,ab.
133. ((patient or personal) adj1 (satisfaction or autonomy or preference$ or experience$)).ti,ab.
134. "activities of daily living".ti,ab.
135. "quality adjusted life year$".ti,ab.
136. (health$ adj1 (life$ or behavio$)).ti,ab.
137. (psycho$ adj1 function$).ti,ab.
138. or/129-137
139. 17 or 21 or 24 or 31 or 43 or 51 or 55 or 59 or 62 or 63 or 66 or 70 or 75 or 86 or 90 or 103 or 109 or 122 or 128 or 138
140. 12 and 139
141. Limit 140 to yr="2010-2021"

PsycINFO

Database and platform: PsycINFO (via OVID) 1806 to present

Search date: 28 May 2021

1. enthesopath$.ti,ab.
2. psoriat$.ti,ab.
3. enthesitis.ti,ab.
4. dactylitis.ti,ab.
5. ((psorias$ or nail$) adj5 (arthrit$ or arthropath$ or monoarthr$ or oligoarthr$ or polyarthr$ or rheumat$)).ti,ab.
6. ((psorias$ or nail$) adj5 (axial or peripheral or spondyl?arthr$ or arthropath$ or spondylitis or sacroili?ti$)).ti,ab.
7. axPsA.ti,ab.
8. pPsA.ti,ab.
9. or/1-8
10. exp Tobacco Smoking/
11. Nicotine/
12. Smoking Cessation/
13. Electronic cigarettes/
14. (smok$ or nicotin$ or tobacco$ or vape or vaping).ti,ab.
15. or/10-14
16. Alcohol Drinking Patterns/
17. Binge Drinking/
18. Alcohol Abuse/
19. Alcoholism/
20. (alcohol$ or booz$ or liquor$).ti,ab.
21. or/16-20
22. exp "Substance Use Disorder"/
23. ((substance$ or drug$ or solvent$ or narcotic$) adj2 (abus$ or depend$ or illegal$ or excess$ or use$ or addict$ or habit$ or usage or misuse or illicit or recreation$)).ti,ab.
24. or/22-23
25. exp Diets/
26. Nutrition/
27. (diet$ or nutriti$).ti,ab.
28. or/25-27
29. Body Mass Index/
30. Obesity/
31. Overweight/
32. Weight loss/
33. Body Weight/
34. (obes$ or bariatri$ or slimm$).ti,ab.
35. (weight$ adj2 (manage$ or health$ or loss$ or gain$ or change$ or over$ or reduct$ or control$ or modific$)).ti,ab.
36. or/29-35
37. exp Exercise/
38. Physical Fitness/
39. Physical Activity/
40. Kinesiology/
41. (exercis$ or kinesiology).ti,ab.
42. (physical$ adj1 (train$ or activit$ or move$ or work$ or exertion or "keep fit")).ti,ab.
43. (train$ adj1 (endurance or resistance or autogenic)).ti,ab.
44. (muscle$ adj1 (stretch$ or strength$ or "work-out$" or "work$ out")).ti,ab.
45. or/37-44
46. exp Anxiety/
47. Mental Health/
48. exp Anxiety Disorders/
49. (anxi$ or "generali?ed anxiety disorder").ti,ab.
50. or/46-49
51. Beck Depression Inventory/
52. "Depression (Emotion)"/
53. Major Depression/
54. Reactive Depression/
55. Zungs Self Rating Depression Scale/
56. Atypical Depression/
57. Endogenous Depression/
58. Late Life Depression/
59. "Long-term Depression (Neuronal)"/
60. Recurrent Depression/
61. Treatment Resistant Depression/
62. exp Affective Disorders/
63. (mood$ adj1 (low$ or reduc$)).ti,ab.
64. (depress$ or miser$ or hopelessness or unhappy or sadness).ti,ab.
65. or/51-64
66. exp Stress/
67. (stress$ or pressure or tension$ or hassle or strain$ or distress or upset$).ti,ab.
68. or/66-67
69. Pain Management/
70. (pain$ adj1 (control$ or relief$ or reduc$ or manage$ or alleviat$ or lesson$)).ti,ab.
71. or/69-70
72. exp Sleep/
73. (sleep$ or rest$ or nap$ or slumber or drows$ or insomnia).ti,ab.
74. or/72-73
75. Fatigue/
76. Sleepiness/
77. (fatigu$ or tired$ or weary or weariness or exhaust$ or lethargy or drain$ or "flare-up$" or "flare up" or flare$).ti,ab.
78. or/75-77
79. Treatment Compliance/
80. (medic$ adj1 ("non-adheren$" or nonadheren$ or persisten$ or "non-complian$" or noncomplian$ or compliance or comply)).ti,ab.
81. (drug$ adj1 ("non-adheren$" or nonadheren$ or persisten$ or "non-complian$" or noncomplian$ or compliance or comply)).ti,ab.
82. or/79-81
83. exp Alternative Medicine/
84. ("complementary medicine" or "complementary therap$").ti,ab.
85. (medicine adj1 (natural$ or folk or holist$ or unconvention$ or alternat$ or herb$ or faith or unorthodox or heal$)).ti,ab.
86. ("traditional Chinese medicine" or "Chinese traditional medicine" or "Chinese herbal medicine").ti,ab.
87. ("alternative therap$" or "faith heal$" or homeopath$ or chiropract$ or osteopath$ or "cranial sacral therapy").ti,ab.
88. ((therap$ or remed$) adj1 (holist$ or herb$)).ti,ab.
89. or/83-88
90. Adjunctive Treatment/
91. ((adjunct$ or adjuvant$) adj1 (therap$ or care or treatment$)).ti,ab.
92. "add-on therapy".ti,ab.
93. or/90-92
94. Physical Therapy/
95. Mind Body Therapy/
96. Cognitive Behavior Therapy/
97. Holistic Health/
98. Massage/
99. Movement Therapy/
100. Aromatherapy/
101. Meditation/
102. Mindfulness/
103. Psycotherapy/
104. Dance Therapy/
105. Music Therapy/
106. Mind Body Therapy/
107. Motivational Interviewing/
108. Cognitive Behavior Therapy/
109. (acupunctur$ or yoga or hypnosis or qigong or meditate$ or meditation or "Tai ji" or "Tai Chi" or Reiki or "mental healing" or psychodrama$ or psychophysiology).ti,ab.
110. ((psycholog$ or psycotherap$) adj1 (biofeedback or imagery)).ti,ab.
111. (exercise$ adj1 breath$).ti,ab.
112. (therap$ adj2 (touch or behavio$ or "acceptance and commitment" or cognitive or animal or dance or exercise or "extracorporeal shockwave" or CBT or "compassion focus$" or psycholog$)).ti,ab.
113. "motivational interview$".ti,ab.
114. (reflexotherapy or reflexology or mindfulness or "cognitive remediation" or "spinal cord stimulation" or "transcutaneous electric nerve stimulation" or TENS or "dry needling").ti,ab.
115. (manipulat$ adj1 (spin$ or osteopath$ or musculoskelet$ or orthop$)).ti,ab.
116. (("non pharmacologic$" or "non-pharmacologic$" or nonpharmacologic$) adj1 (treatment$ or therap$)).ti,ab.
117. (therap$ adj1 (relax$ or massag$ or hydro$ or music$ or physical or physio$ or music$ or spirit$)).ti,ab.
118. "psycholog$ intervention$".ti,ab.
119. or/94-118
120. Psychosexual Behavior/
121. Sex Therapy/
122. Sexual Function Disturbances/
123. intima$.ti,ab.
124. (relation$ adj1 (personal or close$ or caring or familiar$ or affection$)).ti,ab.
125. (emotional$ adj1 (close$ or attachment or together$)).ti,ab.
126. or/120-125
127. Social Isolation/
128. Loneliness/
129. Alienation/
130. Social Psychology/
131. (friend$ or "social relations$" or isolat$ or lonely or loneliness or work or job or employment or career or travel$ or holiday$ or vacation$ or journey$ or excursion$).ti,ab.
132. (relation$ adj3 (friend$ or famil$ or wife$ or husband$ or spouse$ or partner$ or colleague$)).ti,ab.
133. (friend$ adj1 (girl$ or boy$)).ti,ab.
134. or/127-133
135. Professional Referral/
136. Self-Referral/
137. ((health$ or outpatient or clinic) adj1 (attendance or appointment$)).ti,ab.
138. (accept$ adj3 diagnos$).ti,ab.
139. "doctor patient communication".ti,ab.
140. ((consultat$ or communicat$) adj2 (doctor$ or physician$ or medic or medics or nurse$ or physio$ or "healthcare profession$")).ti,ab.
141. or/135-140
142. exp "Quality of Life"/
143. Social Functioning/
144. exp Lifestyle/
145. health behavior/
146. Life Satisfaction/
147. "Activities of Daily Living"/
148. Social Support/
149. "quality of life".ti,ab.
150. (health$ adj1 (life$ or behavio$)).ti,ab.
151. (psycho$ adj1 function$).ti,ab.
152. "life quality".ti,ab.
153. ((patient or personal) adj1 (satisfaction or autonomy or preference$ or experience$)).ti,ab.
154. "activities of daily living".ti,ab.
155. "quality adjusted life year$".ti,ab.
156. or/142-155
157. 15 or 21 or 24 or 28 or 36 or 45 or 50 or 65 or 68 or 71 or 74 or 78 or 82 or 89 or 93 or 119 or 126 or 134 or 141 or 156
158. 9 and 157
159. Limit 158 to yr="2010-2021"
