# Supplementary material for: The impact of psoriatic arthritis on quality of life: a systematic review
Source: Ther Adv Musculoskelet Dis. 2024 Dec 22;16:1759720X241295920. doi: 10.1177/1759720X241295920 (PMC11664531; doi:10.1177/1759720X241295920)
Supplement: sj-docx-3-tab-10.1177_1759720X241295920 – Supplemental material for The impact of psoriatic arthritis on quality of life: a systematic review [file sj-docx-3-tab-10.1177_1759720X241295920.docx]

1. **JBI CRITICAL APPRAISAL CHECKLIST FOR
   ANALYTICAL CROSS-SECTIONAL STUDIES**

Date: 07.04

Author: Azevedo Year: 2019 Record Number: 6567 (Excluded)

|  | Yes | No | Unclear | Not applicable |
| --- | --- | --- | --- | --- |
| 1. Were the criteria for inclusion in the sample clearly defined? | □ | □ | □ | □ |
| 2. Were the study subjects and the setting described in detail? | □ | □ | □ | □ |
| 3. Was the exposure measured in a valid and reliable way? | □ | □ | □ | □ |
| 4. Were objective, standard criteria used for measurement of the condition? | □ | □ | □ | □ |
| 5. Were confounding factors identified? | □ | □ | □ | □ |
| 6. Were strategies to deal with confounding factors stated? | □ | □ | □ | □ |
| 7. Were the outcomes measured in a valid and reliable way? | □ | □ | □ | □ |
| 8. Was appropriate statistical analysis used?  Overall appraisal:               Include     □    **Exclude**     □    Seek further info  □  **Comments (Including reason for exclusion)**   - 2, Background characteristics clear, but setting of study less clear including time period, location, how participants recruited - includes primary outcome but doesn’t include quantitative data data on PsA - Doesn't appear to look at influence of QOL on lifestyle (e.g. diet, weight management) | □ | □ | □ | □ |

1. **JBI CRITICAL APPRAISAL CHECKLIST FOR COHORT STUDIES**

Date: 07.04

Author: Ballegaard Year:2020 Record Number 6567

|  | Yes | No | Unclear | Not applicable |
| --- | --- | --- | --- | --- |
| 1. Were the two groups similar and recruited from the same population? | □ | □ | □ | □ |
| 2. Were the exposures measured similarly to assign people to both exposed and unexposed groups? | □ | □ | □ | □ |
| 3. Was the exposure measured in a valid and reliable way? | □ | □ | □ | □ |
| 4. Were confounding factors identified? | □ | □ | □ | □ |
| 5. Were strategies to deal with confounding factors stated? | □ | □ | □ | □ |
| 6. Were the groups/participants free of the outcome at the start of the study (or at the moment of exposure)? | □ | □ | □ | □ |
| 7. Were the outcomes measured in a valid and reliable way? | □ | □ | □ | □ |
| 8. Was the follow up time reported and sufficient to be long enough for outcomes to occur? | □ | □ | □ | □ |
| 9. Was follow up complete, and if not, were the reasons to loss to follow up described and explored? | □ | □ | □ | □ |
| 10.  Were strategies to address incomplete follow up utilized? | □ | □ | □ | □ |
| 11.  Was appropriate statistical analysis used? | □ | □ | □ | □ |

Overall appraisal:               Include     □    Exclude     □    Seek further info  □

**Comments (Including reason for exclusion)**

- response rates explored, and the data with incomplete response rates not included in follow up analysis

1. **JBI CRITICAL APPRAISAL CHECKLIST FOR
   ANALYTICAL CROSS-SECTIONAL STUDIES**

Date: 07.04

Author: Bandinelli. Year: 2013 Record Number: 6539

|  | Yes | No | Unclear | Not applicable |
| --- | --- | --- | --- | --- |
| 1. Were the criteria for inclusion in the sample clearly defined? | □ | □ | □ | □ |
| 2. Were the study subjects and the setting described in detail? | □ | □ | □ | □ |
| 3. Was the exposure measured in a valid and reliable way? | □ | □ | □ | □ |
| 4. Were objective, standard criteria used for measurement of the condition? | □ | □ | □ | □ |
| 5. Were confounding factors identified? | □ | □ | □ | □ |
| 6. Were strategies to deal with confounding factors stated? | □ | □ | □ | □ |
| 7. Were the outcomes measured in a valid and reliable way? | □ | □ | □ | □ |
| 8. Was appropriate statistical analysis used? | □ | □ | □ | □ |

Overall appraisal:               Include     □    Exclude     □    Seek further info  □

Comments (Including reason for exclusion)

- defined according to CASPAR criteria
- time period not clear. Neither is method of participant selection

1. **JBI CRITICAL APPRAISAL CHECKLIST FOR
   ANALYTICAL CROSS-SECTIONAL STUDIES**

Date: 07.04

Author: Baskan Year: 2016 Record Number: 5923

|  | Yes | No | Unclear | Not applicable |
| --- | --- | --- | --- | --- |
| 1. Were the criteria for inclusion in the sample clearly defined? | □ | □ | □ | □ |
| 2. Were the study subjects and the setting described in detail? | □ | □ | □ | □ |
| 3. Was the exposure measured in a valid and reliable way? | □ | □ | □ | □ |
| 4. Were objective, standard criteria used for measurement of the condition? | □ | □ | □ | □ |
| 5. Were confounding factors identified? | □ | □ | □ | □ |
| 6. Were strategies to deal with confounding factors stated? | □ | □ | □ | □ |
| 7. Were the outcomes measured in a valid and reliable way? | □ | □ | □ | □ |
| 8. Was appropriate statistical analysis used? | □ | □ | □ | □ |

Overall appraisal:               Include     □    Exclude     □    Seek further info  □

Comments (Including reason for exclusion)

- setting clear, but available background characteristics minimal
- Small sample size

1. **JBI CRITICAL APPRAISAL CHECKLIST FOR
   ANALYTICAL CROSS-SECTIONAL STUDIES**

Date: 07.04

Author: Baviere. Year: 2020 Record Number: 5821

|  | Yes | No | Unclear | Not applicable |
| --- | --- | --- | --- | --- |
| 1. Were the criteria for inclusion in the sample clearly defined? | □ | □ | □ | □ |
| 2. Were the study subjects and the setting described in detail? | □ | □ | □ | □ |
| 3. Was the exposure measured in a valid and reliable way? | □ | □ | □ | □ |
| 4. Were objective, standard criteria used for measurement of the condition? | □ | □ | □ | □ |
| 5. Were confounding factors identified? | □ | □ | □ | □ |
| 6. Were strategies to deal with confounding factors stated? | □ | □ | □ | □ |
| 7. Were the outcomes measured in a valid and reliable way? | □ | □ | □ | □ |
| 8. Was appropriate statistical analysis used? | □ | □ | □ | □ |

Overall appraisal:               Include     □    Exclude     □    Seek further info  □

Comments (Including reason for exclusion)

- no time included, when the study was conducted
- use of DAS28-CRP not specific to disease
- no secondary outcomes

1. **JBI CRITICAL APPRAISAL CHECKLIST FOR
   ANALYTICAL CROSS SECTIONAL STUDIES**

Date: 07.04

Author: Billing Year: 2010 Record Number:5643

|  | Yes | No | Unclear | Not applicable |
| --- | --- | --- | --- | --- |
| 1. Were the criteria for inclusion in the sample clearly defined? | □ | □ | □ | □ |
| 2. Were the study subjects and the setting described in detail? | □ | □ | □ | □ |
| 3. Was the exposure measured in a valid and reliable way? | □ | □ | □ | □ |
| 4. Were objective, standard criteria used for measurement of the condition? | □ | □ | □ | □ |
| 5. Were confounding factors identified? | □ | □ | □ | □ |
| 6. Were strategies to deal with confounding factors stated? | □ | □ | □ | □ |
| 7. Were the outcomes measured in a valid and reliable way? | □ | □ | □ | □ |
| 8. Was appropriate statistical analysis used? | □ | □ | □ | □ |

Overall appraisal:               Include     □    Exclude     □    Seek further info  □

Comments (Including reason for exclusion):

not all participants included in follow up included (due to lack of response)

1. **JBI CRITICAL APPRAISAL CHECKLIST FOR
   ANALYTICAL CROSS-SECTIONAL STUDIES**

Date: 08.04

Author: Brihan Year: 2020 Record Number: 5005

|  | Yes | No | Unclear | Not applicable |
| --- | --- | --- | --- | --- |
| 1. Were the criteria for inclusion in the sample clearly defined? | □ | □ | □ | □ |
| 2. Were the study subjects and the setting described in detail? | □ | □ | □ | □ |
| 3. Was the exposure measured in a valid and reliable way? | □ | □ | □ | □ |
| 4. Were objective, standard criteria used for measurement of the condition? | □ | □ | □ | □ |
| 5. Were confounding factors identified? | □ | □ | □ | □ |
| 6. Were strategies to deal with confounding factors stated? | □ | □ | □ | □ |
| 7. Were the outcomes measured in a valid and reliable way? | □ | □ | □ | □ |
| 8. Was appropriate statistical analysis used? | □ | □ | □ | □ |

Overall appraisal:               **Include**     □    Exclude     □    Seek further info  □

Comments (Including reason for exclusion)

- used self-esteem questionnaire rather than QOL limited outcomes and background info
- no healthy control group. experimental group had both PsA and severe Psoriasis, measured outcome of self-esteem could due to both

1. **JBI CRITICAL APPRAISAL CHECKLIST FOR
   ANALYTICAL CROSS SECTIONAL STUDIES**

Date: 08.04

Author: Cano-Garcia Year: 2021. Record Number: 4481

|  | Yes | No | Unclear | Not applicable |
| --- | --- | --- | --- | --- |
| 1. Were the criteria for inclusion in the sample clearly defined? | □ | □ | □ | □ |
| 2. Were the study subjects and the setting described in detail? | □ | □ | □ | □ |
| 3. Was the exposure measured in a valid and reliable way? | □ | □ | □ | □ |
| 4. Were objective, standard criteria used for measurement of the condition? | □ | □ | □ | □ |
| 5. Were confounding factors identified? | □ | □ | □ | □ |
| 6. Were strategies to deal with confounding factors stated? | □ | □ | □ | □ |
| 7. Were the outcomes measured in a valid and reliable way? | □ | □ | □ | □ |
| 8. Was appropriate statistical analysis used? | □ | □ | □ | □ |

Overall appraisal:               Include     □    Exclude     □    Seek further info  □

Comments (Including reason for exclusion)

1. **JBI CRITICAL APPRAISAL CHECKLIST FOR
   ANALYTICAL CROSS SECTIONAL STUDIES**

Date____08.04___________________________

Author__Carneiro 2017___Year_________  Record Number_4927_

|  | Yes | No | Unclear | Not applicable |
| --- | --- | --- | --- | --- |
| 1. Were the criteria for inclusion in the sample clearly defined? | □ | □ | □ | □ |
| 2. Were the study subjects and the setting described in detail? | □ | □ | □ | □ |
| 3. Was the exposure measured in a valid and reliable way? | □ | □ | □ | □ |
| 4. Were objective, standard criteria used for measurement of the condition? | □ | □ | □ | □ |
| 5. Were confounding factors identified? | □ | □ | □ | □ |
| 6. Were strategies to deal with confounding factors stated? | □ | □ | □ | □ |
| 7. Were the outcomes measured in a valid and reliable way? | □ | □ | □ | □ |
| 8. Was appropriate statistical analysis used? | □ | □ | □ | □ |

Overall appraisal:               Include     □    Exclude     □    Seek further info  □

Comments (Including reason for exclusion)

1. **JBI CRITICAL APPRAISAL CHECKLIST FOR
   ANALYTICAL CROSS SECTIONAL STUDIES**

Date____08.04___________________________

Author__Chiowchanwisawakit 2019___Year_________  Record Number_4229_

|  | Yes | No | Unclear | Not applicable |
| --- | --- | --- | --- | --- |
| 1. Were the criteria for inclusion in the sample clearly defined? | □ | □ | □ | □ |
| 2. Were the study subjects and the setting described in detail? | □ | □ | □ | □ |
| 3. Was the exposure measured in a valid and reliable way? | □ | □ | □ | □ |
| 4. Were objective, standard criteria used for measurement of the condition? | □ | □ | □ | □ |
| 5. Were confounding factors identified? | □ | □ | □ | □ |
| 6. Were strategies to deal with confounding factors stated? | □ | □ | □ | □ |
| 7. Were the outcomes measured in a valid and reliable way? | □ | □ | □ | □ |
| 8. Was appropriate statistical analysis used? | □ | □ | □ | □ |

Overall appraisal:               Include     □    Exclude     □    Seek further info  □

Comments (Including reason for exclusion)

1. **JBI CRITICAL APPRAISAL CHECKLIST FOR
   ANALYTICAL CROSS-SECTIONAL STUDIES**

Date:09.04

Author: Coates. Year: 2020 Record Number: 3114

|  | Yes | No | Unclear | Not applicable |
| --- | --- | --- | --- | --- |
| 1. Were the criteria for inclusion in the sample clearly defined? | □ | □ | □ | □ |
| 2. Were the study subjects and the setting described in detail? | □ | □ | □ | □ |
| 3. Was the exposure measured in a valid and reliable way? | □ | □ | □ | □ |
| 4. Were objective, standard criteria used for measurement of the condition? | □ | □ | □ | □ |
| 5. Were confounding factors identified? | □ | □ | □ | □ |
| 6. Were strategies to deal with confounding factors stated? | □ | □ | □ | □ |
| 7. Were the outcomes measured in a valid and reliable way? | □ | □ | □ | □ |
| 8. Was appropriate statistical analysis used? | □ | □ | □ | □ |

Overall appraisal:               Include     □    Exclude     □    Seek further info  □

Comments (Including reason for exclusion)

2- limited comorbidity data. Setting information clear

3/4- NB- point of this survey was patient perspectives. Might be recall bias etc

5- point of paper was hypothesis generating

1. **JBI CRITICAL APPRAISAL CHECKLIST FOR
   ANALYTICAL CROSS-SECTIONAL STUDIES**

Date: 09.04

Author: Conaghan Year: 2020 Record Number: 3640

|  | Yes | No | Unclear | Not applicable |
| --- | --- | --- | --- | --- |
| 1. Were the criteria for inclusion in the sample clearly defined? | □ | □ | □ | □ |
| 2. Were the study subjects and the setting described in detail? | □ | □ | □ | □ |
| 3. Was the exposure measured in a valid and reliable way? | □ | □ | □ | □ |
| 4. Were objective, standard criteria used for measurement of the condition? | □ | □ | □ | □ |
| 5. Were confounding factors identified? | □ | □ | □ | □ |
| 6. Were strategies to deal with confounding factors stated? | □ | □ | □ | □ |
| 7. Were the outcomes measured in a valid and reliable way? | □ | □ | □ | □ |
| 8. Was appropriate statistical analysis used? | □ | □ | □ | □ |

Overall appraisal:               Include     □    Exclude     □    Seek further info  □

Comments (Including reason for exclusion)

outcomes- patient reported/ clinician-based assessment. May be variability in this assessment

1. **JBI CRITICAL APPRAISAL CHECKLIST FOR
   ANALYTICAL CROSS-SECTIONAL STUDIES**

Date: 09.04

Author: Dalal Year:2015 Record Number: 3570

|  | Yes | No | Unclear | Not applicable |
| --- | --- | --- | --- | --- |
| 1. Were the criteria for inclusion in the sample clearly defined? | □ | □ | □ | □ |
| 2. Were the study subjects and the setting described in detail? | □ | □ | □ | □ |
| 3. Was the exposure measured in a valid and reliable way? | □ | □ | □ | □ |
| 4. Were objective, standard criteria used for measurement of the condition? | □ | □ | □ | □ |
| 5. Were confounding factors identified? | □ | □ | □ | □ |
| 6. Were strategies to deal with confounding factors stated? | □ | □ | □ | □ |
| 7. Were the outcomes measured in a valid and reliable way? | □ | □ | □ | □ |
| 8. Was appropriate statistical analysis used? | □ | □ | □ | □ |

Overall appraisal:               Include     □    Exclude     □    Seek further info  □

Comments (Including reason for exclusion)

cofounding comorbidities identified, minimal strategies to deal with these factors

1. **JBI CRITICAL APPRAISAL CHECKLIST FOR COHORT STUDIES**

Date: 09.04

Author: diminno Year: 2013. Record Number: 2167

|  | Yes | No | Unclear | Not applicable |
| --- | --- | --- | --- | --- |
| 1. Were the two groups similar and recruited from the same population? | □ | □ | □ | □ |
| 2. Were the exposures measured similarly to assign people to both exposed and unexposed groups? | □ | □ | □ | □ |
| 3. Was the exposure measured in a valid and reliable way? | □ | □ | □ | □ |
| 4. **Were confounding factors identified?** | □ | □ | □ | □ |
| 5. Were strategies to deal with confounding factors stated? | □ | □ | □ | □ |
| 6. Were the groups/participants free of the outcome at the start of the study (or at the moment of exposure)? | □ | □ | □ | □ |
| 7. Were the outcomes measured in a valid and reliable way? | □ | □ | □ | □ |
| 8. Was the follow up time reported and sufficient to be long enough for outcomes to occur? | □ | □ | □ | □ |
| 9. Was follow up complete, and if not, were the reasons to loss to follow up described and explored? | □ | □ | □ | □ |
| 10.  Were strategies to address incomplete follow up utilized? | □ | □ | □ | □ |
| 11.  Was appropriate statistical analysis used? | □ | □ | □ | □ |

Overall appraisal:               Include     □    Exclude     □    Seek further info  □

Comments (Including reason for exclusion)

1. **JBI CRITICAL APPRAISAL CHECKLIST FOR
   ANALYTICAL CROSS-SECTIONAL STUDIES**

Date: 09.04

Author: Duruoz Year: 2020 Record Number: 2144 (Excluded)

|  | Yes | No | Unclear | Not applicable |
| --- | --- | --- | --- | --- |
| 1. Were the criteria for inclusion in the sample clearly defined? | □ | □ | □ | □ |
| 2. Were the study subjects and the setting described in detail? | □ | □ | □ | □ |
| 3. Was the exposure measured in a valid and reliable way? | □ | □ | □ | □ |
| 4. Were objective, standard criteria used for measurement of the condition? | □ | □ | □ | □ |
| 5. Were confounding factors identified? | □ | □ | □ | □ |
| 6. Were strategies to deal with confounding factors stated? | □ | □ | □ | □ |
| 7. Were the outcomes measured in a valid and reliable way? | □ | □ | □ | □ |
| 8. Was appropriate statistical analysis used? | □ | □ | □ | □ |

Overall appraisal:               Include     □    Exclude     □    Seek further info  □

Comments (Including reason for exclusion)

Not all patients included in all of the outcome measures due to incomplete registry data

1. **JBI CRITICAL APPRAISAL CHECKLIST FOR
   ANALYTICAL CROSS-SECTIONAL STUDIES**

Date: 10.04

Author: Duvetorp Year: 2019 Record Number:2125

|  | Yes | No | Unclear | Not applicable |
| --- | --- | --- | --- | --- |
| 1. Were the criteria for inclusion in the sample clearly defined? | □ | □ | □ | □ |
| 2. Were the study subjects and the setting described in detail? | □ | □ | □ | □ |
| 3. Was the exposure measured in a valid and reliable way? | □ | □ | □ | □ |
| 4. Were objective, standard criteria used for measurement of the condition? | □ | □ | □ | □ |
| 5. Were confounding factors identified? | □ | □ | □ | □ |
| 6. Were strategies to deal with confounding factors stated? | □ | □ | □ | □ |
| 7. Were the outcomes measured in a valid and reliable way? | □ | □ | □ | □ |
| 8. Was appropriate statistical analysis used? | □ | □ | □ | □ |

Overall appraisal:               Include     □    Exclude     □    Seek further info  □

Comments (Including reason for exclusion)

2- background characteristics apparently previously reported, not included in this paper

4- physician reported diagnoses?

7 - patient reported outcomes (reliance on accurate recall of respondant survey)

Seek further info- as no data for just PsA patients

1. **JBI CRITICAL APPRAISAL CHECKLIST FOR COHORT STUDIES**

Date: 10.04

Author: Geijer Year: 2021 Record Number: 7932

|  | Yes | No | Unclear | Not applicable |
| --- | --- | --- | --- | --- |
| 1. Were the two groups similar and recruited from the same population? | □ | □ | □ | □ |
| 2. Were the exposures measured similarly to assign people to both exposed and unexposed groups? | □ | □ | □ | □ |
| 3. Was the exposure measured in a valid and reliable way? | □ | □ | □ | □ |
| 4. Were confounding factors identified? | □ | □ | □ | □ |
| 5. Were strategies to deal with confounding factors stated? | □ | □ | □ | □ |
| 6. Were the groups/participants free of the outcome at the start of the study (or at the moment of exposure)? | □ | □ | □ | □ |
| 7. Were the outcomes measured in a valid and reliable way? | □ | □ | □ | □ |
| 8. Was the follow up time reported and sufficient to be long enough for outcomes to occur? | □ | □ | □ | □ |
| 9. Was follow up complete, and if not, were the reasons to loss to follow up described and explored? | □ | □ | □ | □ |
| 10.  Were strategies to address incomplete follow up utilized? | □ | □ | □ | □ |
| 11.  Was appropriate statistical analysis used? | □ | □ | □ | □ |

Overall appraisal:               Include     □    Exclude     □    Seek further info  □

Comments (Including reason for exclusion)

Not all raw data provided for QOL outcomes

1. **JBI CRITICAL APPRAISAL CHECKLIST FOR
   ANALYTICAL CROSS-SECTIONAL STUDIES**

Date: 10.04

Author: Gezer Year: 2017. Record Number: 8179

|  | Yes | No | Unclear | Not applicable |
| --- | --- | --- | --- | --- |
| 1. Were the criteria for inclusion in the sample clearly defined? | □ | □ | □ | □ |
| 2. Were the study subjects and the setting described in detail? | □ | □ | □ | □ |
| 3. Was the exposure measured in a valid and reliable way? | □ | □ | □ | □ |
| 4. Were objective, standard criteria used for measurement of the condition? | □ | □ | □ | □ |
| 5. Were confounding factors identified? | □ | □ | □ | □ |
| 6. Were strategies to deal with confounding factors stated? | □ | □ | □ | □ |
| 7. Were the outcomes measured in a valid and reliable way? | □ | □ | □ | □ |
| 8. Was appropriate statistical analysis used? | □ | □ | □ | □ |

Overall appraisal:               Include     □    Exclude     □    Seek further info  □

Comments (Including reason for exclusion)

2- minimal background characteristics

1. **JBI CRITICAL APPRAISAL CHECKLIST FOR
   ANALYTICAL CROSS-SECTIONAL STUDIES**

Date: 11.04

Author: Gokmen Year:2014. Record Number: 7506 (Excluded)

|  | Yes | No | Unclear | Not applicable |
| --- | --- | --- | --- | --- |
| 1. Were the criteria for inclusion in the sample clearly defined? | □ | □ | □ | □ |
| 2. Were the study subjects and the setting described in detail? | □ | □ | □ | □ |
| 3. Was the exposure measured in a valid and reliable way? | □ | □ | □ | □ |
| 4. Were objective, standard criteria used for measurement of the condition? | □ | □ | □ | □ |
| 5. Were confounding factors identified? | □ | □ | □ | □ |
| 6. Were strategies to deal with confounding factors stated? | □ | □ | □ | □ |
| 7. Were the outcomes measured in a valid and reliable way? | □ | □ | □ | □ |
| 8. Was appropriate statistical analysis used? | □ | □ | □ | □ |

Overall appraisal:               Include     □    Exclude     □    Seek further info  □

Comments (Including reason for exclusion)

2- minimal setting characteristics- assumed set in Turkey. Not much comorbidity data etc

5- confounding factors not evaluated

1. **JBI CRITICAL APPRAISAL CHECKLIST FOR
   ANALYTICAL CROSS-SECTIONAL STUDIES**

Date: 11.04

Author: Gratacos Year: 2014 Record Number: 7382

|  | Yes | No | Unclear | Not applicable |
| --- | --- | --- | --- | --- |
| 1. Were the criteria for inclusion in the sample clearly defined? | □ | □ | □ | □ |
| 2. Were the study subjects and the setting described in detail? | □ | □ | □ | □ |
| 3. Was the exposure measured in a valid and reliable way? | □ | □ | □ | □ |
| 4. Were objective, standard criteria used for measurement of the condition? | □ | □ | □ | □ |
| 5. Were confounding factors identified? | □ | □ | □ | □ |
| 6. Were strategies to deal with confounding factors stated? | □ | □ | □ | □ |
| 7. Were the outcomes measured in a valid and reliable way? | □ | □ | □ | □ |
| 8. Was appropriate statistical analysis used? | □ | □ | □ | □ |

Overall appraisal:               Include     □    Exclude     □    Seek further info  □

Comments (Including reason for exclusion)

2- minimal setting/ background characteristics. Not much comorbidity data etc

5- confounding factors not evaluated

1. **JBI CRITICAL APPRAISAL CHECKLIST FOR
   ANALYTICAL CROSS-SECTIONAL STUDIES**

Date: 11.04

Author: Gudu. Year: 2016 Record Number: 6870

|  | Yes | No | Unclear | Not applicable |
| --- | --- | --- | --- | --- |
| 1. Were the criteria for inclusion in the sample clearly defined? | □ | □ | □ | □ |
| 2. Were the study subjects and the setting described in detail? | □ | □ | □ | □ |
| 3. Was the exposure measured in a valid and reliable way? | □ | □ | □ | □ |
| 4. Were objective, standard criteria used for measurement of the condition? | □ | □ | □ | □ |
| 5. Were confounding factors identified? | □ | □ | □ | □ |
| 6. Were strategies to deal with confounding factors stated? | □ | □ | □ | □ |
| 7. Were the outcomes measured in a valid and reliable way? | □ | □ | □ | □ |
| 8. Was appropriate statistical analysis used? | □ | □ | □ | □ |

Overall appraisal:               Include     □    Exclude     □    Seek further info  □

Comments (Including reason for exclusion)

2- minimal setting/ background characteristics. Not much comorbidity data etc

5- confounding factors not evaluated

1. **JBI CRITICAL APPRAISAL CHECKLIST FOR
   ANALYTICAL CROSS-SECTIONAL STUDIES**

Date: 11.04

Author: Haugeberg 2020. Year: 2020. Record Number: 13009

|  | Yes | No | Unclear | Not applicable |
| --- | --- | --- | --- | --- |
| 1. Were the criteria for inclusion in the sample clearly defined? | □ | □ | □ | □ |
| 2. Were the study subjects and the setting described in detail? | □ | □ | □ | □ |
| 3. Was the exposure measured in a valid and reliable way? | □ | □ | □ | □ |
| 4. Were objective, standard criteria used for measurement of the condition? | □ | □ | □ | □ |
| 5. Were confounding factors identified? | □ | □ | □ | □ |
| 6. Were strategies to deal with confounding factors stated? | □ | □ | □ | □ |
| 7. Were the outcomes measured in a valid and reliable way? | □ | □ | □ | □ |
| 8. Was appropriate statistical analysis used? | □ | □ | □ | □ |

Overall appraisal:               Include     □    Exclude     □    Seek further info  □

Comments (Including reason for exclusion)

1. **JBI CRITICAL APPRAISAL CHECKLIST FOR
   ANALYTICAL CROSS-SECTIONAL STUDIES**

Date: 11.04

Author: Haugeberg 2020. Year: 2020. Record Number: 13003

|  | Yes | No | Unclear | Not applicable |
| --- | --- | --- | --- | --- |
| 1. Were the criteria for inclusion in the sample clearly defined? | □ | □ | □ | □ |
| 2. Were the study subjects and the setting described in detail? | □ | □ | □ | □ |
| 3. Was the exposure measured in a valid and reliable way? | □ | □ | □ | □ |
| 4. Were objective, standard criteria used for measurement of the condition? | □ | □ | □ | □ |
| 5. Were confounding factors identified? | □ | □ | □ | □ |
| 6. Were strategies to deal with confounding factors stated? | □ | □ | □ | □ |
| 7. Were the outcomes measured in a valid and reliable way? | □ | □ | □ | □ |
| 8. Was appropriate statistical analysis used? | □ | □ | □ | □ |

Overall appraisal:               Include     □    Exclude     □    Seek further info  □

Comments (Including reason for exclusion) minimal confounding factors IDed

1. **JBI CRITICAL APPRAISAL CHECKLIST FOR
   ANALYTICAL CROSS-SECTIONAL STUDIES**

Date: 11.04

Author: Haugeberg 2020. Year: 2020. Record Number: 13000

|  | Yes | No | Unclear | Not applicable |
| --- | --- | --- | --- | --- |
| 1. Were the criteria for inclusion in the sample clearly defined? | □ | □ | □ | □ |
| 2. Were the study subjects and the setting described in detail? | □ | □ | □ | □ |
| 3. Was the exposure measured in a valid and reliable way? | □ | □ | □ | □ |
| 4. Were objective, standard criteria used for measurement of the condition? | □ | □ | □ | □ |
| 5. Were confounding factors identified? | □ | □ | □ | □ |
| 6. Were strategies to deal with confounding factors stated? | □ | □ | □ | □ |
| 7. Were the outcomes measured in a valid and reliable way? | □ | □ | □ | □ |
| 8. Was appropriate statistical analysis used? | □ | □ | □ | □ |

Overall appraisal:               Include     □    Exclude     □    Seek further info  □

Comments (Including reason for exclusion) minimal confounding factors IDed

1. **JBI CRITICAL APPRAISAL CHECKLIST FOR
   CASE CONTROL STUDIES**

Date: 12.04

Author: Hernandez-Hernandez Year: 2018 Record Number: 12757 (Excluded)

|  | Yes | No | Unclear | Not applicable |
| --- | --- | --- | --- | --- |
| 1. Were the groups comparable other than the presence of disease in cases or the absence of disease in controls? | □ | □ | □ | □ |
| 2. Were cases and controls matched appropriately? | □ | □ | □ | □ |
| 3. Were the same criteria used for identification of cases and controls? | □ | □ | □ | □ |
| 4. Was exposure measured in a standard, valid and reliable way? | □ | □ | □ | □ |
| 5. Was exposure measured in the same way for cases and controls? | □ | □ | □ | □ |
| 6. Were confounding factors identified? | □ | □ | □ | □ |
| 7. Were strategies to deal with confounding factors stated? | □ | □ | □ | □ |
| 8. Were outcomes assessed in a standard, valid and reliable way for cases and controls? | □ | □ | □ | □ |
| 9. Was the exposure period of interest long enough to be meaningful? | □ | □ | □ | □ |
| 10.  Was appropriate statistical analysis used? | □ | □ | □ | □ |

Overall appraisal:               Include     □    Exclude     □    Seek further info  □

Comments primary outcomes not met

1. **JBI CRITICAL APPRAISAL CHECKLIST FOR
   ANALYTICAL CROSS-SECTIONAL STUDIES**

Date: 12.04

Author: Howells Year: 2018 Record Number: 12707

|  | Yes | No | Unclear | Not applicable |
| --- | --- | --- | --- | --- |
| 1. Were the criteria for inclusion in the sample clearly defined? | □ | □ | □ | □ |
| 2. Were the study subjects and the setting described in detail? | □ | □ | □ | □ |
| 3. Was the exposure measured in a valid and reliable way? | □ | □ | □ | □ |
| 4. Were objective, standard criteria used for measurement of the condition? | □ | □ | □ | □ |
| 5. Were confounding factors identified? | □ | □ | □ | □ |
| 6. Were strategies to deal with confounding factors stated? | □ | □ | □ | □ |
| 7. Were the outcomes measured in a valid and reliable way? | □ | □ | □ | □ |
| 8. Was appropriate statistical analysis used? | □ | □ | □ | □ |

Overall appraisal:               Include     □    Exclude     □    Seek further info  □

Comments (Including reason for exclusion) minimal confounding factors IDed

1. **JBI CRITICAL APPRAISAL CHECKLIST FOR
   ANALYTICAL CROSS-SECTIONAL STUDIES**

Date: 12.04

Author: Kavanaugh Year: 2016. Record Number: 11175 (Excluded)

|  | Yes | No | Unclear | Not applicable |
| --- | --- | --- | --- | --- |
| 1. Were the criteria for inclusion in the sample clearly defined? | □ | □ | □ | □ |
| 2. Were the study subjects and the setting described in detail? | □ | □ | □ | □ |
| 3. Was the exposure measured in a valid and reliable way? | □ | □ | □ | □ |
| 4. Were objective, standard criteria used for measurement of the condition? | □ | □ | □ | □ |
| 5. Were confounding factors identified? | □ | □ | □ | □ |
| 6. Were strategies to deal with confounding factors stated? | □ | □ | □ | □ |
| 7. Were the outcomes measured in a valid and reliable way? | □ | □ | □ | □ |
| 8. Was appropriate statistical analysis used? | □ | □ | □ | □ |

Overall appraisal:               Include     □    Exclude     □    Seek further info  □

Comments (Including reason for exclusion) outcomes not met, and diagnoses all self reported

1. **JBI CRITICAL APPRAISAL CHECKLIST FOR
   ANALYTICAL CROSS SECTIONAL STUDIES**

Date: 12.04

Author: Kawalec 2016 Year: 2016  Record Number: 11010 (Excluded)

|  | Yes | No | Unclear | Not applicable |
| --- | --- | --- | --- | --- |
| 1. Were the criteria for inclusion in the sample clearly defined? | □ | □ | □ | □ |
| 2. Were the study subjects and the setting described in detail? | □ | □ | □ | □ |
| 3. Was the exposure measured in a valid and reliable way? | □ | □ | □ | □ |
| 4. Were objective, standard criteria used for measurement of the condition? | □ | □ | □ | □ |
| 5. Were confounding factors identified? | □ | □ | □ | □ |
| 6. Were strategies to deal with confounding factors stated? | □ | □ | □ | □ |
| 7. Were the outcomes measured in a valid and reliable way? | □ | □ | □ | □ |
| 8. Was appropriate statistical analysis used? | □ | □ | □ | □ |

Overall appraisal:               Include     □    Exclude     □    Seek further info  □

Comments (Including reason for exclusion)

- minimal background info, or info on confounding factors
- outcome measures not validated, based on other measures,

1. **JBI CRITICAL APPRAISAL CHECKLIST FOR
   ANALYTICAL CROSS-SECTIONAL STUDIES**

Date: 13.04

Author: Kotsis Year: 2012  Record Number:  10304

|  | Yes | No | Unclear | Not applicable |
| --- | --- | --- | --- | --- |
| 1. Were the criteria for inclusion in the sample clearly defined? | □ | □ | □ | □ |
| 2. Were the study subjects and the setting described in detail? | □ | □ | □ | □ |
| 3. Was the exposure measured in a valid and reliable way? | □ | □ | □ | □ |
| 4. Were objective, standard criteria used for measurement of the condition? | □ | □ | □ | □ |
| 5. Were confounding factors identified? | □ | □ | □ | □ |
| 6. Were strategies to deal with confounding factors stated? | □ | □ | □ | □ |
| 7. Were the outcomes measured in a valid and reliable way? | □ | □ | □ | □ |
| 8. Was appropriate statistical analysis used? | □ | □ | □ | □ |

Overall appraisal:               Include     □    Exclude     □    Seek further info  □

Comments (Including reason for exclusion)

1. **JBI CRITICAL APPRAISAL CHECKLIST FOR
   ANALYTICAL CROSS-SECTIONAL STUDIES**

Date: 13.04

Author: Krajewska-Wlodarczyk Year: 2018 Record Number: 10226

|  | Yes | No | Unclear | Not applicable |
| --- | --- | --- | --- | --- |
| 1. Were the criteria for inclusion in the sample clearly defined? | □ | □ | □ | □ |
| 2. Were the study subjects and the setting described in detail? | □ | □ | □ | □ |
| 3. Was the exposure measured in a valid and reliable way? | □ | □ | □ | □ |
| 4. Were objective, standard criteria used for measurement of the condition? | □ | □ | □ | □ |
| 5. Were confounding factors identified? | □ | □ | □ | □ |
| 6. Were strategies to deal with confounding factors stated? | □ | □ | □ | □ |
| 7. Were the outcomes measured in a valid and reliable way? | □ | □ | □ | □ |
| 8. Was appropriate statistical analysis used? | □ | □ | □ | □ |

Overall appraisal:               Include     □    Exclude     □    Seek further info  □

Comments (Including reason for exclusion)

1. Confounding factors not taken into account
2. **JBI CRITICAL APPRAISAL CHECKLIST FOR
   ANALYTICAL CROSS-SECTIONAL STUDIES**

Date: 13.04

Author: Kwan Year: 2019   Record Number: 10335

|  | Yes | No | Unclear | Not applicable |
| --- | --- | --- | --- | --- |
| 1. Were the criteria for inclusion in the sample clearly defined? | □ | □ | □ | □ |
| 2. Were the study subjects and the setting described in detail? | □ | □ | □ | □ |
| 3. Was the exposure measured in a valid and reliable way? | □ | □ | □ | □ |
| 4. Were objective, standard criteria used for measurement of the condition? | □ | □ | □ | □ |
| 5. Were confounding factors identified? | □ | □ | □ | □ |
| 6. Were strategies to deal with confounding factors stated? | □ | □ | □ | □ |
| 7. Were the outcomes measured in a valid and reliable way? | □ | □ | □ | □ |
| 8. Was appropriate statistical analysis used? | □ | □ | □ | □ |

Overall appraisal:               Include     □    Exclude     □    Seek further info  □

Comments (Including reason for exclusion)

some confounding factors, not much in terms of strategies to deal with these.

PROMs measured in relevant QoL domains, which may not be generalisable

1. **JBI CRITICAL APPRAISAL CHECKLIST FOR
   ANALYTICAL CROSS-SECTIONAL STUDIES**

Date: 13.04

Author: Lai Year: 2021 Record Number: 18190

|  | Yes | No | Unclear | Not applicable |
| --- | --- | --- | --- | --- |
| 1. Were the criteria for inclusion in the sample clearly defined? | □ | □ | □ | □ |
| 2. Were the study subjects and the setting described in detail? | □ | □ | □ | □ |
| 3. Was the exposure measured in a valid and reliable way? | □ | □ | □ | □ |
| 4. Were objective, standard criteria used for measurement of the condition? | □ | □ | □ | □ |
| 5. Were confounding factors identified? | □ | □ | □ | □ |
| 6. Were strategies to deal with confounding factors stated? | □ | □ | □ | □ |
| 7. Were the outcomes measured in a valid and reliable way? | □ | □ | □ | □ |
| 8. Was appropriate statistical analysis used? | □ | □ | □ | □ |

Overall appraisal:               Include     □     Exclude     □    Seek further info  □

Comments (Including reason for exclusion) authors acknowledge which confounding factors not included

1. **JBI CRITICAL APPRAISAL CHECKLIST FOR
   ANALYTICAL CROSS-SECTIONAL STUDIES**

Date: 14.04

Author: Lindqvist 2017 Year: 2017   Record Number: 17346

|  | Yes | No | Unclear | Not applicable |
| --- | --- | --- | --- | --- |
| 1. Were the criteria for inclusion in the sample clearly defined? | □ | □ | □ | □ |
| 2. Were the study subjects and the setting described in detail? | □ | □ | □ | □ |
| 3. Was the exposure measured in a valid and reliable way? | □ | □ | □ | □ |
| 4. Were objective, standard criteria used for measurement of the condition? | □ | □ | □ | □ |
| 5. Were confounding factors identified? | □ | □ | □ | □ |
| 6. Were strategies to deal with confounding factors stated? | □ | □ | □ | □ |
| 7. Were the outcomes measured in a valid and reliable way? | □ | □ | □ | □ |
| 8. Was appropriate statistical analysis used? | □ | □ | □ | □ |

Overall appraisal:               Include     □    Exclude     □    Seek further info  □

Comments (Including reason for exclusion)

1- criteria outlined elsewhere referenced. Not much information on comorbidities provided

1. **JBI CRITICAL APPRAISAL CHECKLIST FOR
   ANALYTICAL CROSS-SECTIONAL STUDIES**

Date: 14.04

Author: Mease Year: 2017   Record Number: 15581

|  | Yes | No | Unclear | Not applicable |
| --- | --- | --- | --- | --- |
| 1. Were the criteria for inclusion in the sample clearly defined? | □ | □ | □ | □ |
| 2. Were the study subjects and the setting described in detail? | □ | □ | □ | □ |
| 3. Was the exposure measured in a valid and reliable way? | □ | □ | □ | □ |
| 4. Were objective, standard criteria used for measurement of the condition? | □ | □ | □ | □ |
| 5. Were confounding factors identified? | □ | □ | □ | □ |
| 6. Were strategies to deal with confounding factors stated? | □ | □ | □ | □ |
| 7. Were the outcomes measured in a valid and reliable way? | □ | □ | □ | □ |
| 8. Was appropriate statistical analysis used? | □ | □ | □ | □ |

Overall appraisal:               Include     □    Exclude     □    Seek further info  □

Comments (Including reason for exclusion)

1

1. **JBI CRITICAL APPRAISAL CHECKLIST FOR
   ANALYTICAL CROSS-SECTIONAL STUDIES**

Date: 15.04

Author: Merola Year: 2019. Record Number: 15267

|  | Yes | No | Unclear | Not applicable |
| --- | --- | --- | --- | --- |
| 1. Were the criteria for inclusion in the sample clearly defined? | □ | □ | □ | □ |
| 2. Were the study subjects and the setting described in detail? | □ | □ | □ | □ |
| 3. Was the exposure measured in a valid and reliable way? | □ | □ | □ | □ |
| 4. Were objective, standard criteria used for measurement of the condition? | □ | □ | □ | □ |
| 5. Were confounding factors identified? | □ | □ | □ | □ |
| 6. Were strategies to deal with confounding factors stated? | □ | □ | □ | □ |
| 7. Were the outcomes measured in a valid and reliable way? | □ | □ | □ | □ |
| 8. Was appropriate statistical analysis used? | □ | □ | □ | □ |

Overall appraisal:               Include     □    Exclude     □    Seek further info  □

Comments (Including reason for exclusion)

2- lack of cofounding factors discussion. Also not clear how patients selected to be included into the study.

1. **JBI CRITICAL APPRAISAL CHECKLIST FOR
   ANALYTICAL CROSS-SECTIONAL STUDIES**

 Date: 15.04

Author: Mulder Year: 2021 Record Number: 14644

|  | Yes | No | Unclear | Not applicable |
| --- | --- | --- | --- | --- |
| 1. Were the criteria for inclusion in the sample clearly defined? | □ | □ | □ | □ |
| 2. Were the study subjects and the setting described in detail? | □ | □ | □ | □ |
| 3. Was the exposure measured in a valid and reliable way? | □ | □ | □ | □ |
| 4. Were objective, standard criteria used for measurement of the condition? | □ | □ | □ | □ |
| 5. Were confounding factors identified? | □ | □ | □ | □ |
| 6. Were strategies to deal with confounding factors stated? | □ | □ | □ | □ |
| 7. Were the outcomes measured in a valid and reliable way? | □ | □ | □ | □ |
| 8. Was appropriate statistical analysis used? | □ | □ | □ | □ |

Overall appraisal:               Include     □    Exclude     □    Seek further info  □

Comments (Including reason for exclusion) some background info, not loads. Not clear on PsA diagnostic guidelines

2- lack of cofounding factors discussion. Unclear how patients selected to be included into the study

1. **JBI CRITICAL APPRAISAL CHECKLIST FOR COHORT STUDIES**

Date___14.04____________________________

Author____Orbai_____ Year_2020________  Record Number_20935________

|  | Yes | No | Unclear | Not applicable |
| --- | --- | --- | --- | --- |
| 1. Were the two groups similar and recruited from the same population? | □ | □ | □ | □ |
| 2. Were the exposures measured similarly to assign people to both exposed and unexposed groups? | □ | □ | □ | □ |
| 3. Was the exposure measured in a valid and reliable way? | □ | □ | □ | □ |
| 4. Were confounding factors identified? | □ | □ | □ | □ |
| 5. Were strategies to deal with confounding factors stated? | □ | □ | □ | □ |
| 6. Were the groups/participants free of the outcome at the start of the study (or at the moment of exposure)? | □ | □ | □ | □ |
| 7. Were the outcomes measured in a valid and reliable way? | □ | □ | □ | □ |
| 8. Was the follow up time reported and sufficient to be long enough for outcomes to occur? | □ | □ | □ | □ |
| 9. Was follow up complete, and if not, were the reasons to loss to follow up described and explored? | □ | □ | □ | □ |
| 10.  Were strategies to address incomplete follow up utilized? | □ | □ | □ | □ |
| 11.  Was appropriate statistical analysis used? | □ | □ | □ | □ |

Overall appraisal:               Include     □    Exclude     □    Seek further info  □

Comments (Including reason for exclusion) lack of comorbidity data

1. **JBI CRITICAL APPRAISAL CHECKLIST FOR
   ANALYTICAL CROSS-SECTIONAL STUDIES**

Date: 15.04

Author: Palominos Year: 2020. Record Number: 21080

|  | Yes | No | Unclear | Not applicable |
| --- | --- | --- | --- | --- |
| 1. Were the criteria for inclusion in the sample clearly defined? | □ | □ | □ | □ |
| 2. Were the study subjects and the setting described in detail? | □ | □ | □ | □ |
| 3. Was the exposure measured in a valid and reliable way? | □ | □ | □ | □ |
| 4. Were objective, standard criteria used for measurement of the condition? | □ | □ | □ | □ |
| 5. Were confounding factors identified? | □ | □ | □ | □ |
| 6. Were strategies to deal with confounding factors stated? | □ | □ | □ | □ |
| 7. Were the outcomes measured in a valid and reliable way? | □ | □ | □ | □ |
| 8. Was appropriate statistical analysis used? | □ | □ | □ | □ |

Overall appraisal:               Include     □    Exclude     □    Seek further info  □

Comments (Including reason for exclusion): Not clear on PsA diagnostic guidelines, small sample size

1. **JBI CRITICAL APPRAISAL CHECKLIST FOR
   ANALYTICAL CROSS-SECTIONAL STUDIES**

Date: 15.04

Author: Rodrigues Year: 2019 Record Number: 18708

|  | Yes | No | Unclear | Not applicable |
| --- | --- | --- | --- | --- |
| 1. Were the criteria for inclusion in the sample clearly defined? | □ | □ | □ | □ |
| 2. Were the study subjects and the setting described in detail? | □ | □ | □ | □ |
| 3. Was the exposure measured in a valid and reliable way? | □ | □ | □ | □ |
| 4. Were objective, standard criteria used for measurement of the condition? | □ | □ | □ | □ |
| 5. Were confounding factors identified? | □ | □ | □ | □ |
| 6. Were strategies to deal with confounding factors stated? | □ | □ | □ | □ |
| 7. Were the outcomes measured in a valid and reliable way? | □ | □ | □ | □ |
| 8. Was appropriate statistical analysis used? | □ | □ | □ | □ |

Overall appraisal:               Include     □    Exclude     □    Seek further info  □

Comments (Including reason for exclusion) PsA diagnostic criteria not clear

1. **JBI CRITICAL APPRAISAL CHECKLIST FOR
   ANALYTICAL CROSS-SECTIONAL STUDIES**

Date: 15.04

Author:n Tezel Year: 2015 Record Number: 18708

|  | Yes | No | Unclear | Not applicable |
| --- | --- | --- | --- | --- |
| 1. Were the criteria for inclusion in the sample clearly defined? | □ | □ | □ | □ |
| 2. Were the study subjects and the setting described in detail? | □ | □ | □ | □ |
| 3. Was the exposure measured in a valid and reliable way? | □ | □ | □ | □ |
| 4. Were objective, standard criteria used for measurement of the condition? | □ | □ | □ | □ |
| 5. Were confounding factors identified? | □ | □ | □ | □ |
| 6. Were strategies to deal with confounding factors stated? | □ | □ | □ | □ |
| 7. Were the outcomes measured in a valid and reliable way? | □ | □ | □ | □ |
| 8. Was appropriate statistical analysis used? | □ | □ | □ | □ |

Overall appraisal:               Include     □    Exclude     □    Seek further info  □

Comments (Including reason for exclusion) small sample size

1. **JBI CRITICAL APPRAISAL CHECKLIST FOR
   ANALYTICAL CROSS-SECTIONAL STUDIES**

Date: 15.04

Author: Tillett Year: 2015 Record Number: 21832

|  | Yes | No | Unclear | Not applicable |
| --- | --- | --- | --- | --- |
| 1. Were the criteria for inclusion in the sample clearly defined? | □ | □ | □ | □ |
| 2. Were the study subjects and the setting described in detail? | □ | □ | □ | □ |
| 3. Was the exposure measured in a valid and reliable way? | □ | □ | □ | □ |
| 4. Were objective, standard criteria used for measurement of the condition? | □ | □ | □ | □ |
| 5. Were confounding factors identified? | □ | □ | □ | □ |
| 6. Were strategies to deal with confounding factors stated? | □ | □ | □ | □ |
| 7. Were the outcomes measured in a valid and reliable way? | □ | □ | □ | □ |
| 8. Was appropriate statistical analysis used? | □ | □ | □ | □ |

Overall appraisal:               Include     □    Exclude     □    Seek further info  □

Comments (Including reason for exclusion): lack of confounding factors. Limited on participants baseline characteristics

1. **JBI CRITICAL APPRAISAL CHECKLIST FOR
   ANALYTICAL CROSS SECTIONAL STUDIES**

Date: 16.04

Author: Wervers Year: 2019. Record Number: 25908

|  | Yes | No | Unclear | Not applicable |
| --- | --- | --- | --- | --- |
| 1. Were the criteria for inclusion in the sample clearly defined? | □ | □ | □ | □ |
| 2. Were the study subjects and the setting described in detail? | □ | □ | □ | □ |
| 3. Was the exposure measured in a valid and reliable way? | □ | □ | □ | □ |
| 4. Were objective, standard criteria used for measurement of the condition? | □ | □ | □ | □ |
| 5. Were confounding factors identified? | □ | □ | □ | □ |
| 6. Were strategies to deal with confounding factors stated? | □ | □ | □ | □ |
| 7. Were the outcomes measured in a valid and reliable way? | □ | □ | □ | □ |
| 8. Was appropriate statistical analysis used? | □ | □ | □ | □ |

Overall appraisal:               Include     □    Exclude     □    Seek further info  □

Comments (Including reason for exclusion)

1. **JBI CRITICAL APPRAISAL CHECKLIST FOR COHORT STUDIES**

Date: 16.04

Author: Wong Year: 2021 Record Number: 25723

|  | Yes | No | Unclear | Not applicable |
| --- | --- | --- | --- | --- |
| 1. Were the two groups similar and recruited from the same population? | □ | □ | □ | □ |
| 2. Were the exposures measured similarly to assign people to both exposed and unexposed groups? | □ | □ | □ | □ |
| 3. Was the exposure measured in a valid and reliable way? | □ | □ | □ | □ |
| 4. Were confounding factors identified? | □ | □ | □ | □ |
| 5. Were strategies to deal with confounding factors stated? | □ | □ | □ | □ |
| 6. Were the groups/participants free of the outcome at the start of the study (or at the moment of exposure)? | □ | □ | □ | □ |
| 7. Were the outcomes measured in a valid and reliable way? | □ | □ | □ | □ |
| 8. Was the follow up time reported and sufficient to be long enough for outcomes to occur? | □ | □ | □ | □ |
| 9. Was follow up complete, and if not, were the reasons to loss to follow up described and explored? | □ | □ | □ | □ |
| 10.  Were strategies to address incomplete follow up utilized? | □ | □ | □ | □ |
| 11.  Was appropriate statistical analysis used? | □ | □ | □ | □ |

Overall appraisal:               Include     □    Exclude     □    Seek further info  □

Comments (Including reason for exclusion)

Two groups not necessarily compared so difficult to know if comparable, limitation of QoL assessments
